# An Overview of Naphthylimide as Specific Scaffold for New Drug Discovery

**DOI:** 10.3390/molecules29194529

**Published:** 2024-09-24

**Authors:** Wei Ruan, Zhouling Xie, Ying Wang, Lulu Xia, Yuping Guo, Dan Qiao

**Affiliations:** School of Pharmacy, Jiangxi Science & Technology Normal University, Nanchang 330013, China; ruanwei1216@163.com (W.R.); xiezhouling2021@163.com (Z.X.); 13257976927@163.com (Y.W.); 17839837110@163.com (L.X.)

**Keywords:** naphtylimide, antibacterial, antifungal, anticancer, antiviral, biological activity

## Abstract

Naphthylimides play a pivotal role as aromatic heterocyclic compounds, serving as the foundational structures for numerous pharmacologically significant drugs. These drugs encompass antibacterial, antifungal, anticancer, antimalarial, antiviral, anti-inflammatory, antithrombotic, and antiprotozoal agents. The planar and heteroaromatic characteristics of naphthylimides grant them a strong ability to intercalate into DNA. This intercalation property renders naphthylimide derivatives highly valuable for various biological activities. The advantageous pharmacological activity and ease of synthesis associated with naphthylimides and their derivatives provide significant benefits in the design and development of new compounds within this class. Currently, only a few such molecules are undergoing preclinical and clinical evaluations. In this paper, we have compiled the literature on naphthylimides reported by researchers from 2006 to 2024. Our focus lies on exploring the pharmacological activities of their analogues from a drug development and discovery perspective, while examining their structure–activity relationship and mechanisms of action.

## 1. Introduction

Heterocyclic compounds play a pivotal role in the fields of organic and medicinal chemistry, serving as a crucial bridge between life sciences and biochemistry [1,2]. The diverse range of heterocyclic compounds offers a vast chemical space for exploring their potential medicinal properties [3,4]. In recent years, researchers have directed their attention towards scaffold structures containing heterocyclic atoms and rings [5,6,7,8].

**Figure 1:**
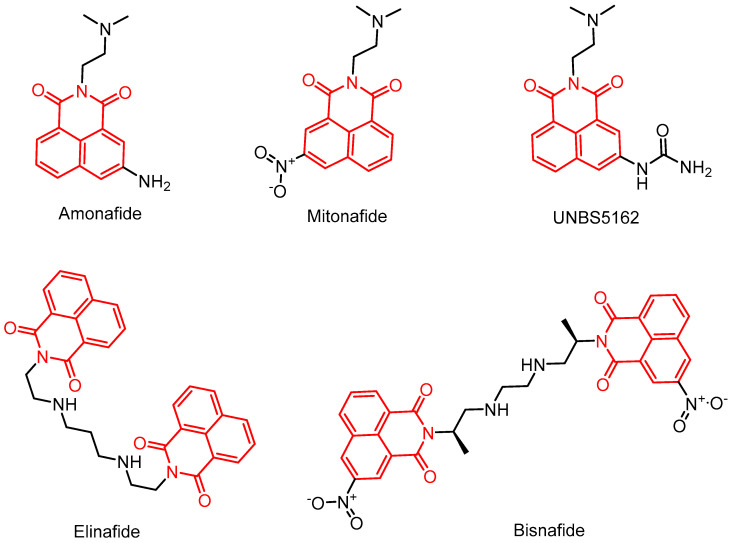
Structures of compounds with heterocyclic naphthylimine backbones already in clinical development trials (The red part is the structure of naphthylimide).

Naphthylimides (syn. naphthalimides or naphthimides) are a significant class of nitrogen-containing aromatic heterocycles, comprising a cyclic diimide and a naphthalene skeleton with the key structural features of 1H-benzisoquinoline-1,3-(2H)-dione [9]. Naphthimide derivatives with conjugated rigid structures typically exhibit excellent fluorescence properties [10]. Furthermore, they possess the capability to interact with biologically active sites such as deoxyribonucleic acid (DNA), topoisomerase (TOP), and human serum albumin (HSA) in biological systems [11]. Various weak binding bonds, including hydrogen bonds, ligand bonds, π-π stacking interactions, hydrophobic interactions, and van der Waals forces, hold potential for diverse medical applications, such as encompass the treatment of various diseases, as well as the diagnosis and detection of life-important illnesses [12,13]. So far, a number of compounds containing a heterocyclic naphthimide backbone, such as Amonafide, Mitonafide, UNBS5162, Elinafide, Bisnafide, and Bibenilone (Figure 1), have been developed for clinical trials. Both Mitonafide and Amonafide are capable of binding to double-stranded DNA through intercalation and can induce topoisomerase II (topo II)-mediated DNA cleavage at nucleotide 1830 of pBR322 DNA [11], confirming the significant potential of naphthylimides for medical applications within the fields of antitumor, antibacterial, and antifungal treatments. Research on naphthylimide-based compounds has emerged as a prominent global area of interest, with extensive investigations aimed at exploring their multi-targeting capabilities in order to develop broad-spectrum drugs that are highly potent, resistant to low levels of resistance, exhibit low toxicity, and possess high bioavailability.

**Figure 2:**
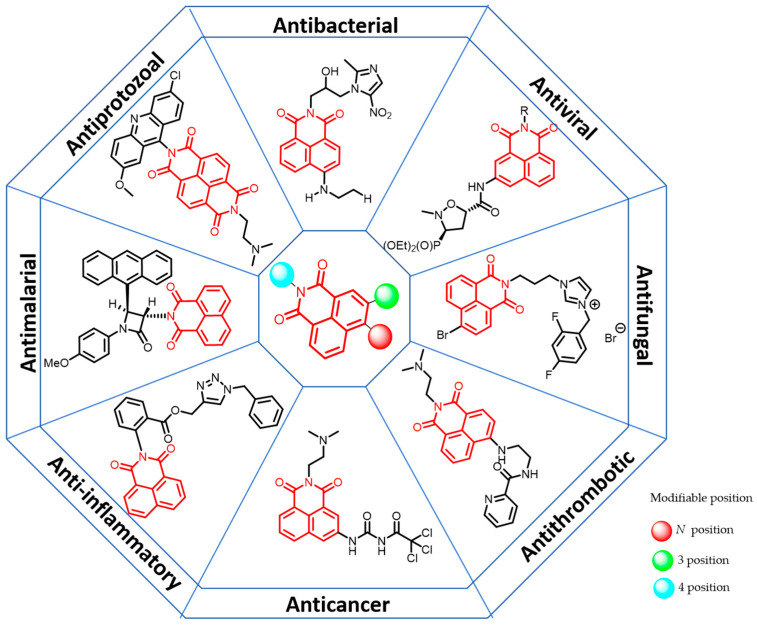
Examples of naphthylimide-containing drugs and their pharmacological applications.

The favorable pharmacological activity and ease of synthesis of naphthylimides and their derivatives are significant advantages for the design and development of novel naphthylimides and their affixes. This paper presents a comprehensive review of recent advancements in antibacterial, antifungal, anticancer, antimalarial, and antiviral studies of naphthimide-containing heteroconjugates (Figure 2). The review will delve into their mechanism of action, key aspects of design, and practical applications. Relevant articles from 2006 to 2024 were identified through a thorough search and screening of the Web of Science and PubMed databases.

## 2. Progress in Biological Activity of Naphthylimide

### 2.1. Antimicrobial Activity

Antimicrobial resistance (AMR) is considered one of the most serious threats to global public health, posing a significant risk to human health and social development [14]. The efficacy of antibacterial drugs is limited by the rapid development of resistance in target bacteria, leading to severe drug resistance in both Gram-positive and Gram-negative bacteria [15,16]. Few new antimicrobial drugs have been discovered worldwide, leading to a situation where clinically used antimicrobial drugs are no longer effective in treating infectious diseases caused by drug-resistant bacteria [17]. As a result, the development of antimicrobial drugs with novel structures and mechanisms of action remains a significant challenge for scientists. Naphthylimide compounds have emerged as promising new structural backbones for antimicrobial agents, being frequently reported and showing good potential.

As a newly emerging class of DNA-targeted chemotherapeutic antimicrobial skeletons, naphthylimide fragments have large structural backbones similar to those of established variant triggers [16]. The incorporation of active fragments into the naphthylimide core is anticipated to produce potent antimicrobial agents and alternative variant triggers [18]. Based on the study of the structure–activity relationship (SAR) of numerous 1,8-naphthimide derivatives, it has been found that changes in the *N*-position and 4-positions of the naphthimide moiety have a significant effect on antimicrobial efficacy [19]. Furthermore, numerous structures containing naphthimide fragments have demonstrated the potential to induce the generation and accumulation of reactive oxygen species (ROS) in DNA damage pathways. This characteristic would significantly enhance their effectiveness in antimicrobial applications [20,21].

#### 2.1.1. Triazole-Containing Naphthylimide Hybrids

Heterocyclic triazoles have received increasing attention due to their significant antimicrobial potential, especially as antifungal drugs for treating infectious diseases [22]. Some triazoles, such as fluconazole and voriconazole are widely utilized as antifungal drugs in clinical settings. However, the extensive use of these drugs has led to the development of drug resistance, which has significantly impacted their therapeutic effectiveness [23].

**Figure 3:**
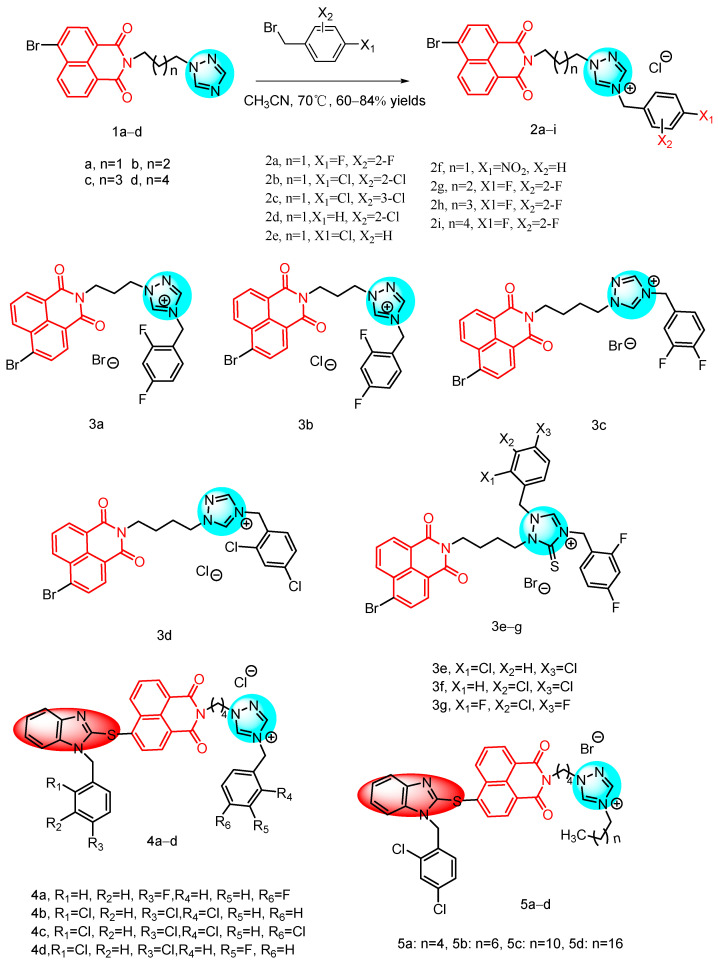
Chemical structures of Naphthalimide containing triazole hybrids **1a**–**d**, **2a**–**i**, **3a**–**g**, **4a**–**d**, **5a**–**d**.

In 2011, Zhang et al. [24] reported a novel series of naphthylimide azole compounds, which included naphthalene imide-derived triazoles **1a**–**d** and **2a**–**i** (Figure 3). The inhibitory activity of these compounds was evaluated in vitro see Table 1 below. Compounds **2a**–**i** exhibited potent activity against all tested strains, with MIC values ranging from 1 to 32 µg/mL. In contrast, the precursor triazoles **1a**–**d** showed low susceptibility, even at high concentrations. Furthermore, compounds **2a**–**f**, derived from the quaternization of (CH_2_)_3_-linked triazole **1a** using differently substituted benzyl halides, demonstrated significant antimicrobial effects. Specifically, the 2,4-difluorobenzene and 2,4-dichlorobenzene derivatives **2a** and **2b** had comparable or better activity than the control drugs chloramphenicol and ofloxacin against all tested bacteria, except *Staphylococcus aureus*, at concentrations of 1 to 8 µg/mL. Notably, compound **2a**, containing a 2,4-difluorophenyl group, showed the strongest inhibitory effect against *Pseudomonas aeruginosa*, being 16-fold stronger than chloramphenicol and comparable to obifloxacin. It is also noteworthy that compound **2a** had good anti-MRSA activity (MIC = 4 µg/mL), comparable to chloramphenicol and orbifloxacin. This result suggests that the presence of a fluorine atom is particularly important in medicine, as it is highly lipophilic and aids in the biotransport and distribution of compounds. Selective synthesis of 2,4-difluorobenzotriazoles with different alkyl linkers produced compounds **2g**–**i**, which showed good bacteriostatic properties (MIC = 2–32 µg/mL). Particularly noteworthy are compounds **2g** and **2h**, which demonstrated potent bacteriostatic properties. Notably, compound **2g** exhibited an eight-fold superiority over chloramphenicol against *Pseudomonas aeruginosa*, with a minimum inhibitory concentration (MIC) of 2 µg/mL. These findings corroborate that the azolium moiety is effective in impeding bacterial proliferation. Additionally, the study suggested that the antimicrobial efficacy of triazolium derivatives bridged by (CH_2_)_3_ and (CH_2_)_4_ groups is enhanced in comparison to their counterparts bridged by (CH_2_)_5_ and (CH_2_)_6_ groups.

In 2013, Guri L. V. et al. [21] reported a series of naphthylimide triazole analogs by incorporating the naphthylimide *N*-position with a five-membered triazole ring (Figure 3). These derivatives were evaluated for their activity against eight Gram-positive and Gram-negative bacteria, as well as two fungal strains see Table 1 below, using chloramphenicol, orbifloxacin, and fluconazole as control drugs. The majority of the derivatives exhibited comparable or superior antimicrobial activity compared to the control drugs. Structure–activity relationship (SAR) analysis revealed that triazoles **3a**–**d** with (CH_2_)_3_ or (CH_2_)_4_ linkers displayed enhanced antimicrobial activity and a broader spectrum than their counterparts. Notably, the 2,4-difluorophenyl derivatives of triazoles **3a** and **3c** demonstrated particularly potent activity against MRSA, *Bacillus subtilis*, *Bacillus luteus*, *Pseudomonas aeruginosa*, and *S. typhi*, with potencies comparable to or greater than orbifloxacin and chloramphenicol. Furthermore, thiotriazoles **3e**–**g** not only surpassed triazoles **3a**, 3**b** in terms of antimicrobial activity but also exhibited potency comparable to or even better than clinical drugs. Thiotriazoles **3f** and **3g**, containing 3,4-dichlorophenyl and 2,4-difluorophenyl groups, respectively, exhibited potent inhibition against all tested bacteria. Compounds **3a** and **3b** demonstrated particularly strong activity against *Bacillus luteus* and *S. typhimurium*, with MIC values of 2 μg/mL, comparable to or superior to chloramphenicol and orbifloxacin as well as clinical drugs. The antifungal findings indicated that certain naphthylimino-triazole derivatives **3a**–**g** displayed promising efficacy against *Candida albicans* and other *Candida species*. Consistent with the bacteriostatic results, the MIC values ranged from 1 to 32 μg/mL for the naphthylimino-triazole derivatives. These compounds exhibited a broad spectrum of antimicrobial activity by effectively inhibiting both Gram-positive and Gram-negative bacteria, including drug-resistant MRSA, while also demonstrating significant antifungal potential.

In 2015, Luo et al. [20] reported a series of benzimidazole-derived naphthylimide triazoles **4a**–**d**, **5a**–**d** (Figure 3), and evaluated their in vitro antibacterial activity using a 2-fold serial dilution method see Table 1 below. Triazole **4a**, containing a 4-fluorophenyl fragment, exhibited potent antimicrobial activity with a broad spectrum and MIC values ranging from 14 to 29 μg/mL. Replacement of the 4-fluorophenyl with a 2,4-dichlorobenzene fragment resulted in compound **4c**, which demonstrated effective inhibitory activity at 7 μg/mL against *Bacillus subtilis*. Both the 2-chlorobenzotriazole derivative **4b** and the octyl-containing compound **5b** showed significant inhibitory activity against *Staphylococcus aureus*, with compound **5b** displaying the most potent inhibitory concentration at only 2 μg/mL. This was comparable to norfloxacin (MIC = 2 μg/mL) and more potent than chloramphenicol (MIC = 7 μg/mL). Compound **5b** exhibited a broad-spectrum antimicrobial effect, with MIC values ranging from 2 to 29 μg/mL. Its anti-*Staphylococcus aureus* and anti-*Bacillus subtilis* activities (MIC = 2 and 4 μg/mL, respectively) were found to be superior to chloramphenicol. Triazolines **4b** and **4d**, containing 3-fluorophenyl fragments, demonstrated the most potent activity against all tested fungal strains (MIC = 2–19 μg/mL). Further fluorescence and UV-visible spectroscopic studies revealed that compound **4b** effectively intercalated into calf thymus DNA to form a complex, inhibiting DNA replication and exerting a robust antimicrobial effect. Benzimidazole-modified naphthylimide triazole derivatives displayed a broad antimicrobial spectrum, demonstrating strong bacteriostatic and antifungal activities. Therefore, these novel benzimidazole naphthylimide triazoles hold promise for further development.

**Figure 4:**
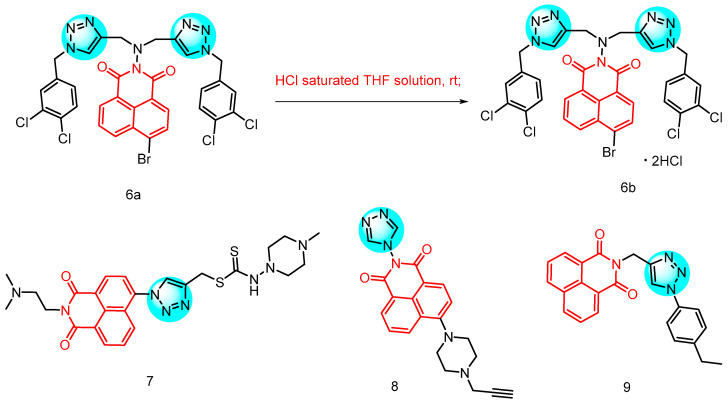
Chemical structures of Naphthalimide containing triazole hybrids **6a**,**b**, **7**–**9**.

In 2013, Lv et al. [25] reported a series of novel antimicrobial derivatives of 1,2,3-triazolylnaphthamide (Figure 4). The synthesized compounds underwent evaluation for their in vitro antibacterial activity against a range of bacterial and fungal species see Table 1 below. The bioactivity assay demonstrated that compound **6a** and its hydrochloride **6b**, particularly the former, exhibited superior antimicrobial activity against *Escherichia coli* compared to norfloxacin and chloramphenicol. Initial studies indicated that compound **6a** effectively interacted with calf thymus DNA to form a complex, thereby inhibiting DNA replication and exerting bacteriostatic effects. Furthermore, human serum albumin was found to efficiently bind and transport compound **6a** through electrostatic interactions.

In 2020, Chen et al. [26] reported 15 novel dithiocarbamate derived naphthoimides, all of which exhibited bacteriostatic activity against *Candida albicans*, *Escherichia coli*, *Bacillus subtilis* and *Staphylococcus aureus*. Compound **7** (Figure 4) demonstrated potent inhibitory effects against *Bacillus subtilis* with a minimum inhibitory concentration of 7.6 µM. Furthermore, the conformational analysis revealed that the incorporation of a triazole ring contributed to enhanced activity of Naphthylimide derivatives, while specific substituents at the 4-position of the naphthoimides displayed increased potency.

In 2021, Zhang et al. [27] reported the discovery of a novel class of Naphthylimine triazole derivatives based on naphthalic anhydride. Among these molecules, the propargyl piperazine derivative **8** (Figure 4) exhibited rapid antifungal activity, low toxicity to normal cells, and no drug resistance. Furthermore, it was found to form supramolecular complexes with DNA, inhibiting its normal function, as well as to interact with CYP51B enzyme to hinder sterol synthesis on the cell membrane. Compound **8** also induced the production and accumulation of ROS, leading to oxidative damage and ultimately cell death. These findings highlight the multi-targeted synergistic antifungal effects of these naphthyliminotriazole molecules against *Aspergillus fumigatus* and their potential as potent antifungal agents.

In 2023, Yadav et al. [28] reported 28 Phthalimide/Naphthalimide containing 1,2,3-triazole hybrids through the click reaction of perterminal alkynes with aromatic azides. The inhibitory activity of the synthesized 1,2,3-triazoles against 2 Gram-positive bacteria, 2 Gram-negative bacteria and 2 fungi was evaluated, revealing moderate to good activity for most compounds. Compound **9** (Figure 4), which contains naphthylimide in its structure, showed a MIC of 0.0163 μmol/mL against both *Klebsiella pneumoniae* and *Candida albicans*, with an inhibitory effect comparable to that of the standard drug fluconazole.

#### 2.1.2. Triazine-Containing Naphthylimide Hybrids

**Figure 5:**
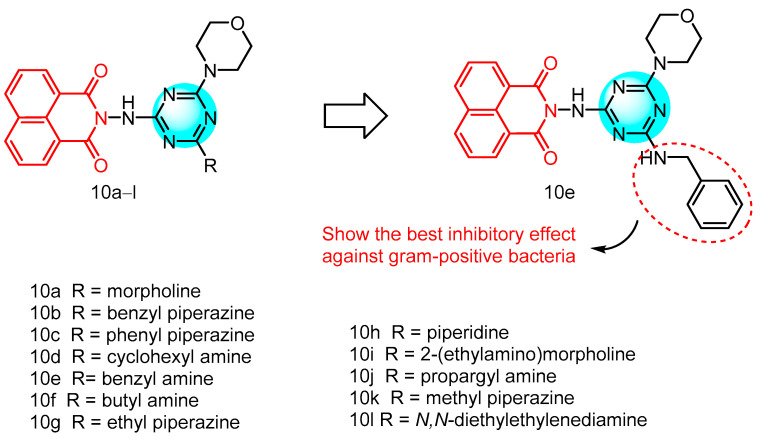
Chemical structures of compounds **10a**–**i**.

In the recent years, the 1,3,5-triazine moiety has emerged as a pivotal scaffold in the realm of materials science, owing to its superior properties. In 2023, Gupta et al. [29] reported a novel series of compounds, which employed triazine, naphthylimine, and lignin as their structural frameworks (Figure 5), and assessed their antibacterial efficacy see Table 1 below. Notably, several compounds exhibited greater potency than the reference antibiotics (chloramphenicol and amoxicillin) in impeding bacterial growth. Specifically, compounds **10a** and **10b** demonstrated minimum inhibitory concentrations (MIC) of 1.56 and 6.25 µg/mL, respectively, against *Staphylococcus aureus* and *Listeria* sp. Similarly, compounds **10c** and **10d** matched this potency, inhibiting the growth of both *Staphylococcus aureus* and *Listeria* sp. at MIC values of 1.56 and 6.25 µg/mL, respectively. Moreover, compounds **10c** and **10d** effectively curtailed the growth of *Listeria* sp. within a concentration range of 0.39 µg/mL to 3.12 µg/mL, whereas compound **10f** showcased an impressive MIC value of 0.09 µg/mL against *Enterococcus faecalis*. Compound **10g**, containing ethyl piperazine, demonstrated selective inhibition of Listeria monocytogenes growth with an MIC value of 3.12 µg/mL. Derivatives **10h**, **10i**, and **10**j exhibited varying levels of activity against the tested strains. These compounds showed potent inhibition against *Staphylococcus aureus*, *Listeria monocytogenes*, and *Enterobacter faecalis* with MIC values of 1.56 µg/mL, 0.048 µg/mL, and 0.048 µg/mL respectively. Compounds **10k** and **10l** displayed reduced MIC values against *Listeria monocytogenes* at 0.006 and 0.19 µg/mL respectively. Compound **10e**, an aniline-substituted triazine, exhibited significant impact on the growth of bacterial strains. It demonstrated superior efficacy against all Gram-positive bacteria, with MIC values ranging from 0.003 to 1.56 µg/mL, outperforming commercially available drugs such as amoxicillin and chloramphenicol. Notably, any substitution on the aniline benzene ring led to a loss of biological activity, while complete loss of activity was observed when naphthylimine was substituted, highlighting its essential role in the antimicrobial activity of the compounds. Mechanistic studies revealed that compound **10e** disrupts cell membranes leading to leakage of intracellular proteins and inhibits biofilm formation in *S. aureus* within 11 days, thereby preventing bacterial resistance. Furthermore, it effectively binds to HSA with a binding constant of 1.32 × 10^5^ M^−1^ for rapid delivery to the target site and hinders bacterial colonization through DNA-binding ability. These findings suggest that compound **10e** holds promise as a potential antimicrobial agent worthy of further development.

#### 2.1.3. Naphthylimide-Derived Metronidazole

**Figure 6:**
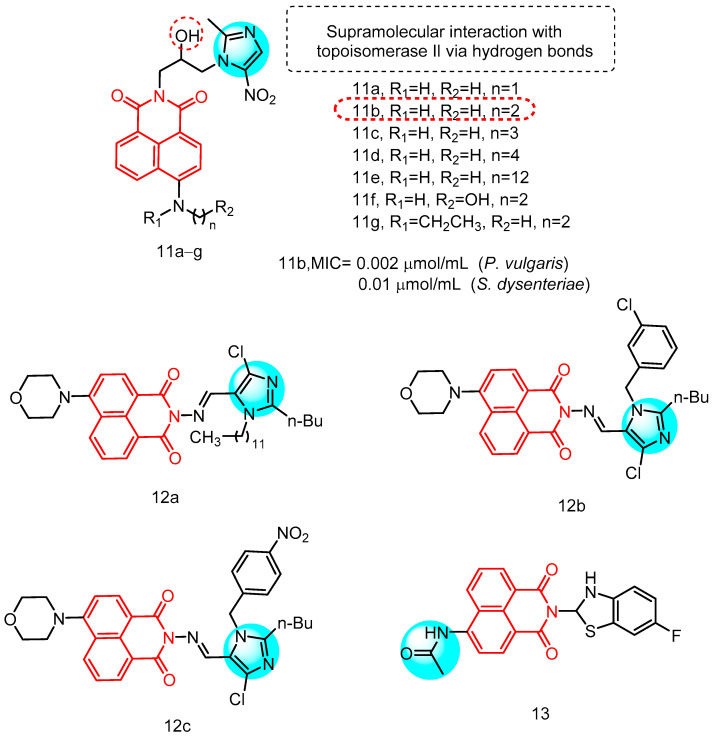
Chemical structures of compounds **11a**–**g**, **12a**–**c**, **13**.

Metronidazole is a widely utilized clinical agent for the treatment of infectious diseases and demonstrates a broad spectrum of biological activities, particularly in terms of antibacterial activity [30]. In 2017, Kang et al. [31] reported a series of novel naphthylimide-derived metronidazoles for the first time (Figure 6). It has been documented that reactive intermediates formed by nitro reduction in microorganisms can covalently bind to DNA and elicit adverse reactions. Stereoprotection of the nitro group is essential to enhance metabolic and physicochemical properties. Therefore, they introduced a substantial structural fragment to facilitate stereoprotection of nitro groups through reduction, thereby generating active intermediates in microorganisms while avoiding triggering adverse reactions and overcoming drug resistance. Nine derivatives **11a**–**g** were evaluated for their antimicrobial activity against a range of Gram-positive bacteria, Gram-negative bacteria, and fungal strains, including methicillin-resistant *Staphylococcus aureus* (MRSA) see Table 1 below. The majority of the compounds exhibited moderate to good antimicrobial activity in vitro. Notably, ethylamine derivative **11b** demonstrated superior broad-spectrum antibacterial efficacy compared to the other compounds. Compound **11b** displayed the highest potency among the tested derivatives with a MIC value of 0.002 µmol/mL, surpassing metronidazole, chloramphenicol, and norfloxacin by 95-fold, 50-fold, and 10-fold respectively. Furthermore, *E. dysenteriae* and *E. coli* (DH52) showed greater sensitivity to compound **11b** (MIC = 0.01 µmol/mL and 0.04 µmol/mL) than to chloramphenicol (MIC = 0.05 µmol/mL and 0.10 µmol/mL). Compound **11b** exhibited superior activity compared to chloramphenicol against MRSA, *S. aureus* and *S. typhi*. Particularly noteworthy is the 5-fold increase in inhibitory potency against MRSA compared to metronidazole, with a MIC value of 0.04 µmol/mL. These findings suggest that naphthylimine metronidazole **11b** holds significant promise for the development of more effective broad-spectrum antimicrobial agents. Furthermore, SAR analysis revealed that the amino group positively influenced biological activity, and the length of the alkyl chain also impacted antimicrobial efficacy, with suitable alkyl chains favoring biological activity of the target compounds. However, introducing hydroxyl groups on the alkyl chain in compound **11f** did not enhance inhibitory efficacy relative to compound **11b**. Similarly, ethylation of the secondary amino group in compound **11g** did not result in increased inhibitory potency; although its MIC value against *S. typhi* was higher than that of chloramphenicol (MIC = 0.10 µmol/mL). The most active molecule, **11b**, not only formed a stable supramolecular complex with calf thymus DNA to inhibit DNA replication and exert antimicrobial activity but also efficiently interacted with HSA through formation of a biosupramolecular complex at a ratio of 1:1. This suggests that naphthylimidomethylnitrozole has great potential for development as a potent broad-spectrum antimicrobial agent.

#### 2.1.4. Schiff BASE-Linked Imidazolylnaphthimide

In 2016 Gong et al. [32] reported a series of Schiff base-conjugated imidazole naphthylimide compounds (Figure 6) for their in vitro antimicrobial activity against a panel of four Gram-positive bacteria, six Gram-negative bacteria, and five fungi see Table 1 below. The majority of the compounds exhibited varying degrees of antimicrobial activity against the tested strains in vitro. Notably, compound **12a** demonstrated a MIC value of 0.01 µmol/mL against *Escherichia coli* (JM109), which is ten times more potent than the clinical drug chloramphenicol and shows potential for overcoming resistance. Additionally, chlorobenzene derivative **12b** effectively inhibited the growth of *Bacillus subtilis* at a concentration of 0.007 µmol/mL, surpassing chloramphenicol by fourteen-fold and comparable to norfloxacin. Furthermore, nitrobenzene compound **12c** displayed the highest inhibitory potency against MRSA with a MIC value of 0.003 µmol/mL, 17-fold stronger than chloramphenicol and 7-fold stronger than norfloxacin, suggesting its promise as an anti-MRSA drug candidate. Additionally, naphthylimine **12c** exhibited moderate activity against other tested strains. Despite having the same anti-MRSA MIC as the currently reported compound **12c**, structural DGLV, in comparison to compound **12c**, should be considered more suitable as a lead compound for novel anti-MRSA drugs due to its facile synthesis, flexibility and lower molecular weight. The antifungal results indicated that the majority of the target compounds demonstrated similar inhibitory tendencies and strengths in their antimicrobial activity.

#### 2.1.5. Naphthylimides and Allylenes

In 2013, Kumari et al. [33] reported a straightforward and efficient approach for synthesizing naphthylimine and allylidene derivatives, resulting in the synthesis of 11 derivatives (Figure 6) which were subsequently evaluated for their inhibitory activities against a range of bacteria using the broth dilution method. The MIC values of all compounds fell within the concentration range of 0.65 to 80 µg/mL. Notably, Compound **13** (MIC < 0.65 µg/mL), featuring a cycloimide structure with an acetamide bond linking *N*-position at the 6-position, exhibited superior inhibitory activity comparable to that of ciprofloxacin and vancomycin. The potential mechanism underlying the antibacterial activity of these compounds may involve interaction with bacterial topoisomerases I and II.

#### 2.1.6. Novel Naphthylimide Aminothiazole

The emergence and dissemination of multidrug-resistant bacteria due to the overuse and misuse of antibiotics poses a significant obstacle to the effective treatment of infectious diseases. Methicillin-resistant *Staphylococcus aureus* (MRSA) [34,35], currently a major hospital pathogen, originated from methicillin-sensitive *Staphylococcus aureus* acquiring the Staphylococcal cassette chromosome mec (SCCmec) through horizontal gene transfer. SCCmec expresses an altered penicillin-binding protein, PBP2a, with low binding affinity for almost all available *β*-lactam antibiotics, rendering them ineffective against MRSA [36,37]. The altered interaction of the naphthylimide analog aminothiazole with PBP2a induces active site exposure and leads to the resurgence of old drugs [38,39].

**Figure 7:**
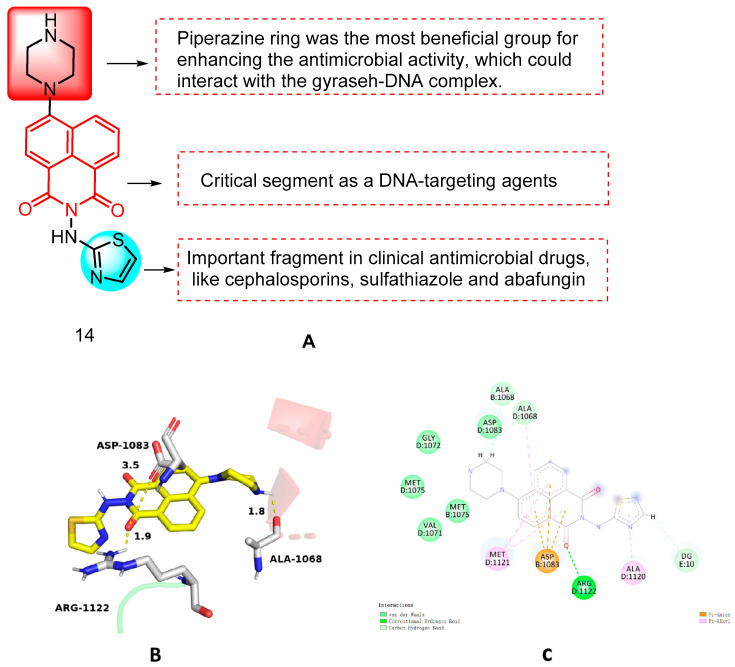
(**A**) Chemical structures of compound **14** and summary of the structure–activity relationships for naphthalimide aminothiazoles. (**B**) Three-dimensional conformation of compound **14** docked in the gyrase−DNA complex (PDB code: 2XCS), Yellow dotted lines indicate hydrogen bonding interactions. (**C**) Two-dimensional conformation of compound 14 docked in the gyrase−DNA complex (PDB code: 2XCS).

In 2017, Chen et al. [38] reported a series of novel naphthimide-aminothiazole hybrids and evaluated their efficacy against a panel of five Gram-positive bacteria, six Gram-negative bacteria, and five fungi using the 2-fold dilution method. Among these compounds, piperazinyl derivative **14** (Figure 7) exhibited a broad spectrum of antimicrobial activity. Structure–activity relationship (SAR) analysis revealed that the presence of alicyclic amino substituents significantly influenced its inhibitory activity. Furthermore, it was found that the piperazinyl ring could interact with the gyraseh–DNA complex, thereby enhancing its antimicrobial potency. Conversely, the alkyl group on the piperazine ring was found to be detrimental to its antimicrobial activity. Additionally, modification at the 4-position of the aminothiazole ring was also observed to have an adverse effect on its antimicrobial potential. Notably, compound **14** demonstrated effective inhibition against MRSA and *Escherichia coli*, with minimum inhibitory concentration (MIC) values of 4 µg/mL and 8 µg/mL, respectively. Compound **14** exhibited membrane activity, low toxicity on mammalian cells, and rapid MRSA eradication, without eliciting bacterial resistance. Molecular docking studies showed that the docking pattern of compound **14** with the gyrase–DNA complex (PDB code: 2XCS) was as shown in Figure 7. Molecule **14** can interact with ARG-1122 of the gyrase–DNA complex through the formation of a hydrogen bond between the carbonyl group of the naphthylimide backbone and the DNA base pair. The unbonded p orbitals on the amino group of ASP-1083 are able to interact with the π-electronic system of the naphthylimide ring, which results in a p-π conjugation effect. This effect enhances the mutual attraction between the two and contributes to the stability of the complex, which plays an important role in the binding ability of the ligand and the stability of the docked conformation. In addition, the NH group of the piperazine ring at the 4h position of the naphthylimide fragment is very close to residue ALA-1068, which indicates the necessity of the NH group for enhancing the biological activity. The binding behavior suggests that the interaction between HSA and the **14** molecule is spontaneous, with hydrophobic interactions and hydrogen bonding playing crucial roles in the translocation of derivative **14**. Therefore, naphthyliminothiazole **14** may serve as a promising multi-targeted anti-MRSA drug candidate.

**Figure 8:**
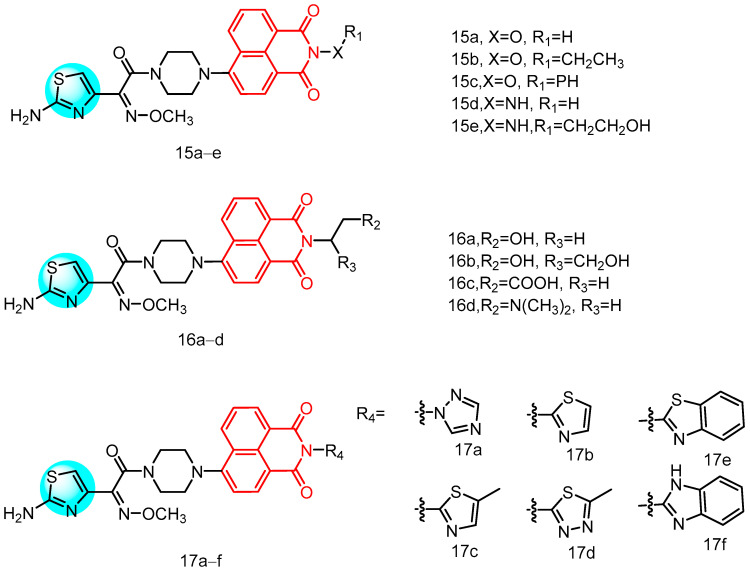
Chemical structures of compounds **15a**–**e**, **16a**–**d**, and **17a**–**f**.

In 2021, Zhang et al. [39] reported a series of novel compounds (Figure 8) characterized by the integration of an aminothiazole oxime moiety and a piperazine bridge at the 4-position of the naphthylimide core, along with substitution at the *N*-position with various substituents. These compounds were evaluated for their activity against five Gram-positive and six Gram-negative bacteria see Table 1 below, revealing that almost all target compounds containing an aminothiazole oxime moiety exhibited stronger inhibitory activity compared to the non-aminothiazole oxime-containing fragments of the intermediates. The naphthimide-based aminothiazole oximes, particularly those with longer alkyl chains or larger hydrophobic alicyclic rings, exhibited significant advantages in anti-MRSA activity. SAR analysis revealed that the incorporation of a heteroatom linker at the *N*-position of the naphthimide portion disrupted the affinity equilibrium, leading to loss of bioactivity. Furthermore, the presence of the heteratomic linker efficiently enhanced the antimicrobial activity, especially against MRSA. Notably, derivatives **15a** (hydroxy), **15b** (ethoxy), **15c** (phenoxy), **15d** (amino), and **15e** (hydroxyethylamine) demonstrated superior or equal anti-MRSA activities compared to norfloxacin. In addition to the enhanced anti-MRSA activity, there was also a noticeable enhancement in the antibacterial activity against other bacteria, possibly attributed to the increased likelihood of heteroatom linkers interacting with biomolecules and promoting the overall antibacterial efficacy. Among compounds **16a**–**d** containing ethyl units linking hydrophilic groups, the dimeglumine group (**16d**) exhibited the most potent anti-MRSA effect, with an MIC value of 0.5 µg/mL, which was 2-fold higher than tetracycline, 4-fold higher than vancomycin, 8-fold higher than ciprofloxacin, and 32-fold higher than norfloxacin. Furthermore, compound **16d** demonstrated broad-spectrum inhibitory activity against reference drugs such as MRSA, *S. aureus* ATCC 25,923, *S. aureus* ATCC 29,213, *Enterococcus faecalis*, *Klebsiella pneumoniae*, *E. coli* ATCC 25,922, and *P. aeruginosa* ATCC 27,853, suggesting that naphthylimide modified by dimethylenediamine segments has significant potential for development. Notably, compound **16d** not only exhibits bactericidal potential but also functions as an antimicrobial agent by triggering a PBP2a metabotropic tensile reaction and acting as a mutagen to revitalize old drugs.

In summary, these findings suggest that derivative **16d** has the potential to inhibit MRSA, including strains with low PBP2a affinity for cefotaxime. The mutagenic inhibition of PBP2a presents a promising therapeutic avenue for further investigation of naphthylimine-based aminothiazole oximes. Furthermore, compound **16d** appears to exert its antimicrobial effects on MRSA cells through multiple mechanisms, including membrane damage, protein leakage, LDH inhibition, and induction of oxidative stress. Additionally, the introduction of azoles and benzazoles onto naphthylimine-based aminothiazole oximes demonstrates a similar trend in enhancing anti-MRSA activity within this series. Notable among these derivatives is **17d,** which exhibits significant anti-MRSA activity with an MIC value as low as 1 µg/mL, surpassing many tested reference drugs. Moreover, triazole derivative **17a**, methylthiazole derivative **17c**, and benzothiazole derivative **17e** also display notable sensitivity against *S. aureus* ATCC 29,213 or *S. aureus* inhibition compared to certain reference drugs. The presence of azoles in various clinical antimicrobial agents underscores their facilitation of antimicrobial activity. Overall, these medicinal chemical biology studies highlight the potential for development of novel naphthylimine-based aminothiazole oximes as specific antimicrobial agents.

#### 2.1.7. Naphthylimide Derivatives Containing Propylene Glycol

**Figure 9:**
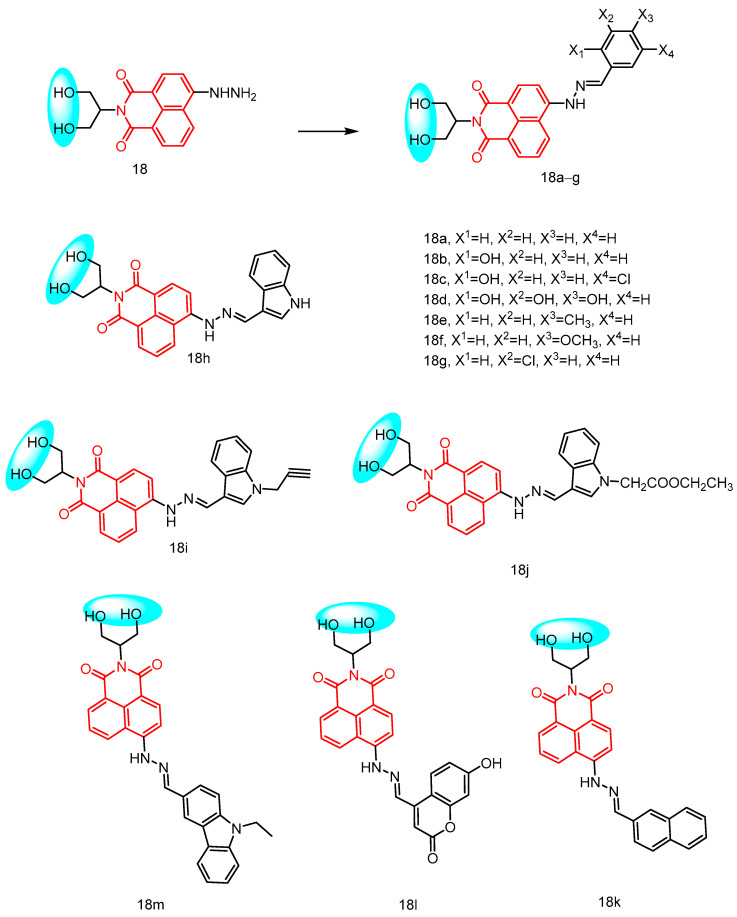
Chemical structures of compounds **18a**–**m**.

In 2022, Zhang et al. [40] reported a new class of structured naphthyliminopropanediols (NIOLs) with potential broad-spectrum antimicrobial activity. They incorporated the 1,3-propanediol fragment and hydrazone fragment into the *N*-position and 4-position of the naphthimide core, respectively, and structurally modified the NIOLs on the appendages connecting the hydrazone segments (Figure 9) to generate a series of compounds for evaluating their antimicrobial effects. Following structural optimizations, it was observed that doping of the six-membered ring enhanced antimicrobial activity, while compound **18a** containing a benzene ring exhibited increased antibacterial activity against *P. aeruginosa* and *S. aureus* ATCC 25,923, being 4-fold and 8-fold more active than norfloxacin, respectively. This enhancement is attributed to the modification of the benzene ring which improved antibacterial efficacy. Several derivatives exhibited potent antimicrobial activity, with compound **18b** containing phenol demonstrating a 2-fold and 8-fold increase in activity against MRSA compared to ciprofloxacin and norfloxacin, respectively. Compound **18c** containing the 4-chloro-o-phenol fragment displayed superior inhibitory activity (MIC = 1–2 μg/mL) against MRSA, *S. aureus* ATCC 25,923, and *P. aeruginosa* strains. Additionally, trihydroxyphenyl compound **18d** showed improved inhibitory activity (MIC = 2 μg/mL) against *S. aureus*, *Klebsiella pneumoniae*, and *P. aeruginosa* ATCC 27,853 strains with good inhibitory activity (MIC = 2–4 μg/mL). The introduction of p-methylphenyl in compound **18e** reduced the MIC of *S. aureus* to 0.5 μg/mL, which was four times lower than that of ciprofloxacin and sixteen times lower than that of norfloxacin. Furthermore, p-Methoxyphenyl-containing compound **18f** exhibited decreased activity against Gram-positive bacteria but demonstrated significant potency against *P. aeruginosa* (MIC = 0.5 μg/mL), which was eight times higher than that of norfloxacin. Lastly, the minimal MIC value for m-chlorophenyl compound **18g** against *S. aureus* was found to be at an impressive level of only 0.25 μg/mL, surpassing all tested reference drugs. Furthermore, the inclusion of diverse larger structural rings also augmented the antimicrobial efficacy. Indole **18h** exhibited a MIC value of 2 μg/mL against *S. aureus* ATCC 29,213, while propargyl derivatives **18i** (MIC = 0.5 μg/mL) and *N*-(2-ethoxy-2-oxoethyl)-indol-3-yl derivative **18j** (MIC = 0.25 μg/mL) demonstrated promising anti-S-A activity. Additionally, 3-Naphthyl **18k** (MIC = 0.5 μg/mL) displayed superior anti-P-A activity compared to norfloxacin (MIC = 4 μg/mL), and compound **l8l** exhibited potent anti-*P. aeruginosa* activity against *S. aureus*, *K. pneumonia*, *S. aureus* ATCC 29,213, and *S. aureus* ATCC 25,923. The introduction of *N*-ethylcarbazol-3-yl fragment significantly enhanced the inhibitory activity of compound **l8m** against *S. aureus*, *S. aureus* ATCC 29,213, and *E. coli* bacteria (MIC = 0.25–0.5 μg/mL). Mechanistic investigations into these highly active compounds revealed that oxidative damage was notably intensified by the accumulation of ROS and disruption of antioxidant defense systems. Meanwhile, novel naphthyliminopropylene glycol derivatives have demonstrated the ability to target and disrupt the membranes of *S. aureus* or *P. aeruginosa*, as evidenced by membrane depolarization, inner and outer membrane permeability, and leakage of intracellular substances. Furthermore, these membrane-targeting compounds exhibit efficacy in eradicating biofilms formed by *S. aureus* or *P. aeruginosa* and effectively killing strains within the biofilm. Additionally, it is worth noting that these derivatives possess the advantage of overcoming drug resistance and hold potential as broad-spectrum antimicrobial agents, thus warranting further development.

#### 2.1.8. Novel Naphthylimide-Thiourea Derivatives

**Figure 10:**
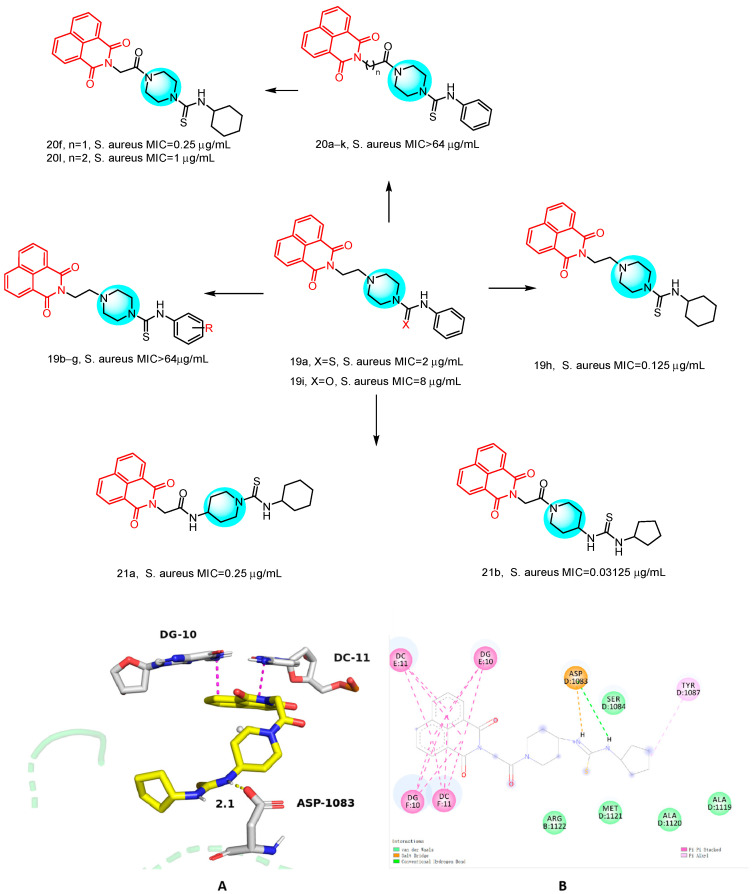
Chemical structures of compounds **19a**–**i**, **20a**–**k**, and **21a**,**b,** and binding interactions of compound **21b** at the active site of *S. aureus* DNA gyrase (PDBid-2XCS), Yellow dotted lines indicate hydrogen bonding interactions, and purple dotted lines indicate π-π stacking interactions. (**A**) Three-dimensional representation of compound **21b**, (**B**) 2D representation of compound **21b**.

In 2024, Rana et al. [41] reported a series of novel naphthylimide-thiourea derivatives (Figure 10), which were subjected to ESKAP assay and Mycobacterium pathogen assay. The synthesized compounds were evaluated for their bacteriostatic activity see Table 1 below, and it was found that compound **21b** showed the most potent bacteriostatic activity against *S. aureus* (MIC = 0.03125 μg/mL). Compound **19h** also showed inhibitory activity against *S. aureus* (MIC = 0.125 μg/mL). Compound **21b** was non-toxic to Vero cells, with an SI value >3200. Additionally, compound **21b** demonstrated equivalent activity against MDR *S. aureus* (including VRSA), with an MIC of 0.06 μg/mL. Moreover, compound **21b** exhibited concentration-dependent bacteriostatic properties. Molecular docking studies indicated that compound **21b** exerts antibacterial activity by inhibiting the DNA gyrase enzyme. To understand the binding mode and interactions of compound **21b**, we performed molecular docking of the active site of *S. aureus* DNA gyrase complexed with GSK299423 (PDB ID 2XCS) as shown in Figure 10. The resultant compound formed a hydrogen bond at **21b** with ASP-1083 at a distance of 2.1 Å via a thiourea-based nitrogen atom. The naphthimide ring of the compound was DNA intercalated with DG10 and DC11 through π-π stacking interactions, as shown in Figure 10, which revealed the stability of compound **21b** at the active site and its positive interactions over a simulation period of 100 ns. Furthermore, Rana et al. through ADME studies confirmed that the compounds adhere to Lipinski’s rule of five and are considered druggable. These findings suggest that these compounds hold potential for further development as potent antimicrobial agents.

#### 2.1.9. Norfloxacin-Substituted Naphthyl Imide Derivatives

**Figure 11:**
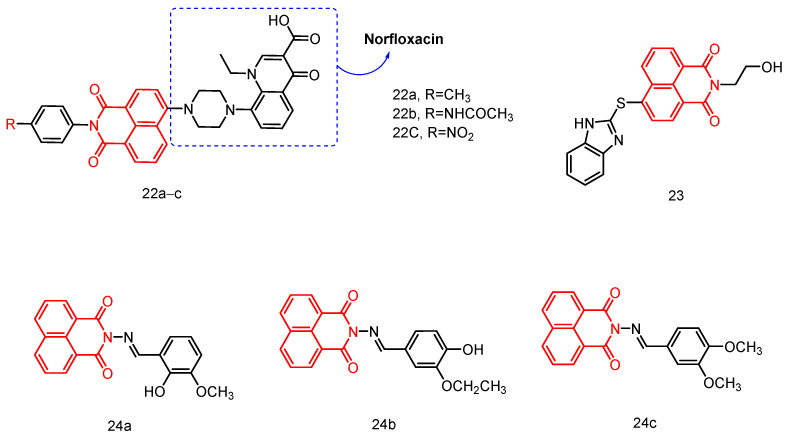
Chemical structures of compounds **22a**–**c**, **23**, and **24a**–**c**.

In 2018, Kumar et al. [42] reported the synthesis of a norfloxacin-substituted naphthylimine derivative (Figure 11) and conducted molecular docking simulations to evaluate its fluorescence properties and antibacterial activity. These derivatives, along with neobiotin, a well-established ATP site inhibitor, were subjected to molecular docking at the ATP binding sites of type II topoisomerase rotamase B and ParE. Compound **22a** exhibited high to moderate affinity for the ATP site and formed numerous hydrogen bonds compared to neobiotin. Competitive inhibition assays were subsequently performed to validate this hypothesis and assess the biological activity of the compounds. The results demonstrated that norfloxacin-substituted naphthylimine derivatives possessed favorable fluorescence, antimicrobial activity, and biological properties, positioning them as potential drug candidates.

#### 2.1.10. Hydroxyethyl Naphthimide

In 2022, Zhang et al. [43] reported a family of hydroxyethyl naphthimides with synergistic chemical and dynamic antifungal effects, demonstrating significant antifungal activity against *A. fumigatus*, *Fusarium tropicalis*, *C. tropicalis*, and *C. parapsilosis* ATCC 22,019. Among these compounds, thioether benzimidazole compound **23** (Figure 11) exhibited superior DNA binding capacity and anti-*C. properties* compared to fluconazole, while also displaying low cytotoxicity, minimal hemolysis, and no significant resistance. Furthermore, compound **23** demonstrated strong lipase affinity and the ability to penetrate cell membranes, causing dysfunction. Mechanistic studies guided by ROS and RNIs revealed that membrane lipid peroxidation and oxidation of GSH to GSSG significantly enhanced intracellular oxidative damage in *C. tropicalis*, disrupting its antioxidant defense system and leading to cell death. Additionally, the combined involvement of chemical and dynamic antifungal treatments disrupted the biological functions of DNA and CPR in *Fusarium tropicalis*, resulting in metabolic inactivation for potential development as specific antifungal drugs based on promising chemical biology studies related to hydroxyethyl naphthylimides.

#### 2.1.11. Naphthalimide-Based Schiff Base Compounds

In 2015, Nayab et al. [44] reported a series of novel naphthylimido Schiff base compounds with potential as DNA binding agents (Figure 11), antioxidants, and antimicrobial agents. The DNA binding properties of the target compounds to Ct-DNA (calf thymus) were thoroughly investigated using various biophysical techniques, including UV-visible spectroscopy, fluorescence spectroscopy, ethidium bromide displacement assay, time-resolved fluorescence spectroscopy, viscosity measurements, cyclic voltammetry, and circular dichroism. The evidence suggested that the test compounds can interact with DNA through insertional binding. Molecular docking results further supported the insertional binding of the test compounds to DNA. The calculated binding energies of the docked compounds (**24a**–**c**) ranged from −8.20 to −8.69 kcal/mol, indicating a strong binding affinity to CtDNA. The synthesized compounds exhibited promising antibacterial activity against *Escherichia coli*, *Staphylococcus aureus*, *Klebsiella pneumoniae*, and *Salmonella typhimurium*. Compound **24c** demonstrated the most potent inhibitory effect against all four bacteria, with MIC values ranging from 0.031 to 0.062 µg/mL. Furthermore, mutagenicity studies revealed that all tested compounds were non-mutagenic in both the presence and absence of metabolic activation. Additionally, antioxidant activity assays indicated the potential scavenging activity of these compounds against DPPH and H_2_O_2_ radicals.

#### 2.1.12. Naphthalimide–Coumarin Hybrids

**Figure 12:**
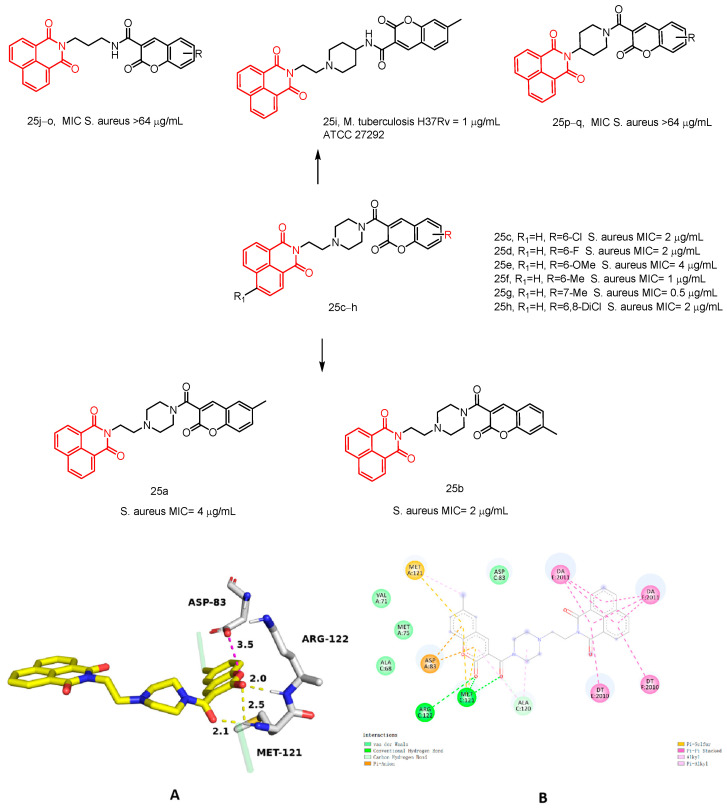
Chemical structures of compounds **25a**–**q** and binding pose of compound **25f** at the active site of *S. aureus* DNA gyrase (PDBid-6QTK), Yellow dotted lines indicate hydrogen bonding interactions, and purple dotted lines indicate ionic interaction. (**A**) Three-dimensional representation of compound **25f**, (**B**) 2D representation of compound **25f**.

In 2024, Rana et al. [45] reported a series of novel naphthylimide–coumarin hybrids **25a**–**q** (Figure 12) and evaluated their inhibitory activity against *Escherichia coli*, *Staphylococcus aureus*, Staphylococcus epidermidis, Klebsiella pneumoniae, *Pseudomonas aeruginosa,* and Mycobacterium tuberculosis H37Rv see Table 1 below. Among the synthesized compounds, compounds **25a**–**h** exhibited potent inhibitory activity against *Staphylococcus aureus* ATCC 29,213, with MIC values ranging from 0.5 to 4 µg/mL. These compounds demonstrated low toxicity to Vero cells (IC_50_ > 50) and a good selectivity index (SI > 12.5). Compounds **25a**–**h** also displayed strong inhibitory effects against a wide range of multidrug-resistant strains, with MIC values ranging from 0.5 to 16 µg/mL for vancomycin-resistant *Staphylococcus aureus* (VRSA). Furthermore, compounds **25f** and **25g** exhibited progressive inhibition of *Staphylococcus aureus* growth for up to 6 h without any antagonism towards FDA-approved drugs. Notably, compound **25i** demonstrated potent bacteriostatic activity (MIC = 1 µg/mL) against Mycobacterium tuberculosis H37Rv, while also displaying non-toxicity to Vero cells with a favorable selectivity index. Additionally, it showed strong efficacy against drug-resistant TB strains. To understand the binding mode and interaction of compound **25f**, we performed docking studies of the compound at the active site of the *S. aureus* DNA–gyrase complex, Gepotidacin (PDB ID 6QTK). Gepotidacin inhibits ASP-83 through the formation of an ionic interaction between piperidinamine and ASP-83 at a distance of 3.5 Å. This interaction is a key factor in the inhibition of the *Staphylococcus aureus* DNA splicase. The nitrogen of the pyranopyridine ring formed water-mediated hydrogen bonds with ASP-83, ARG-122, and MET-121, indicating that the protein conformation was stabilized, as shown in Figure 12. In addition, Rana et al. confirmed that its druggability was within acceptable parameters by ADME analysis. Taken together, these findings suggest that this naphthylimide–coumarin hybrid holds promise for further development in pharmaceutical research and development endeavors.

### 2.2. Naphthylimide Dendrimers with Antimicrobial Activity

Dendrimers are intriguing polymer structures known for their monodispersity and perfect branching symmetry [46]. They possess a combination of polymer and macromolecule properties, and are rich in closely related functional groups that can be modified to enhance their activity and expand the application areas of new material [47], with 1,8-naphthimide derivatives playing a significant role. The color and intensity of fluorescence emitted by these derivatives can be controlled by altering the electron donor capacity of the substituent group at the C-4 position of the chromophore system. This feature makes them widely applicable in designing sensor systems for detecting metal ions and acids in both environmental and biological settings [48]. Additionally, 1,8-naphthalimide derivatives, as part of cyclic imides with a large π-fused skeleton, have demonstrated strong biomedical interest, due to their ability to interact with various biological systems through non-covalent interactions such as π-π stacking. These novel compounds have shown promising microbiological activities, including antibacterial, antiviral, antifungal, anti-inflammatory, and antitumor effects [49,50].

#### 2.2.1. Polypropylamine Dendritic Macromolecules

**Figure 13:**
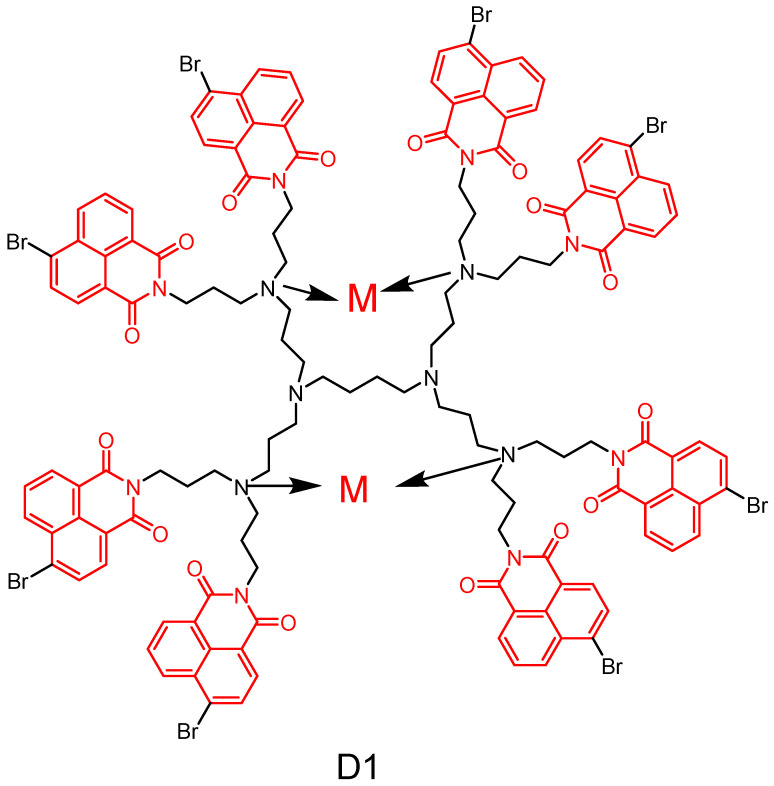
Possible complexation of metal ions with dendrimer **D1**.

In 2014, Staneva et al. [51] reported the synthesis of a novel second-generation polypropylamine dendritic macromolecule (Figure 13) containing eight 4-bromo-1,8-naphthimide units and their zinc and copper complexes at the periphery of the dendritic macromolecule. The photophysical properties of the metal-ion-free dendritic polymer and its complexes were investigated in organic solvents with varying polarities. In vitro antimicrobial screening demonstrated that the newly synthesized metal dendritic macromolecules exhibited potent bacteriostatic activity against several pathogenic Gram-positive and Gram-negative bacteria, as well as *Saccharomyces cerevisiae* and pathogenic *C. cerevisiae*. Furthermore, it was observed that the microbial activity of the complexes remained unaffected by the nature of the metal ions present. These findings suggest that these new metal complexes have potential for developing novel antimicrobial formulations to effectively combat infections caused primarily by *Fusobacterium*, *Micrococcus*, and *Candida*.

**Figure 14:**
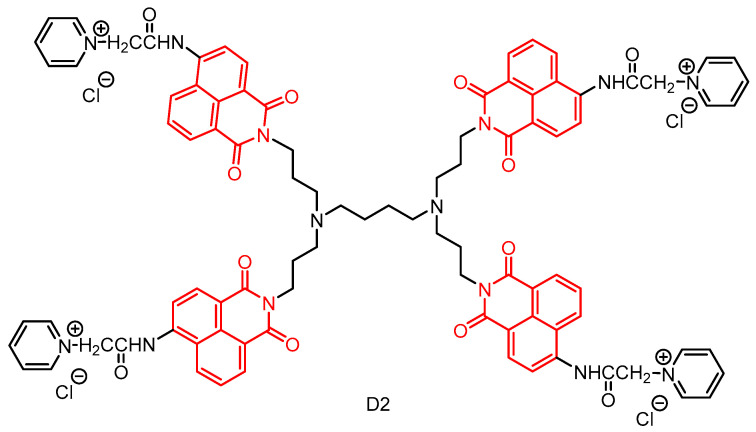
Chemical structures of water-soluble cationic dendrimer **D2**.

In 2020, Staneva et al. [52] reported a water-soluble cationic dendrimer by modifying a first-generation polypropylene imine dendrimer with 1,8-naphthylimide in a three-step process (Figure 14). The photophysical properties of the novel dendrimer were investigated in organic solvents with varying polarities and in aqueous solutions, revealing its absorption and emission of blue fluorescence in the UV region. Furthermore, the bacteriostatic activity of **D2** was evaluated against Gram-positive, Gram-negative, and *Candida species.* The results demonstrated that **D2** exhibited strong bacteriostatic activity against *Bacillus cereus* and moderate activity against *Pseudomonas aeruginosa*. Notably, when deposited on cotton fabrics, the antimicrobial activity of **D2** remained unchanged and was particularly effective against *Bacillus cereus*. These findings suggest that the newly developed dendritic macromolecule **D2** holds promise for potential applications in designing and producing antimicrobial textiles with controlled release of bioactives from cotton matrices.

**Figure 15:**
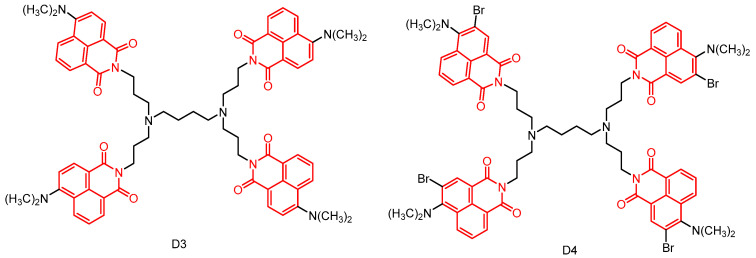
Chemical structures of photoactive dendrimers **D3** and **D4**.

In 2020, Staneva et al. [53] reported two new photoactive dendrimers through peripheral modification of first-generation polypropylene imine dendrimers (Figure 15). The photophysical properties of all compounds were investigated in ethanol and chloroform, revealing their absorption and emission of yellow–green fluorescence in the visible region. Furthermore, their antimicrobial activity in solution and after deposition on cotton fabrics was examined. Notably, their study represents the first investigation into the effect of light on the antibacterial activity of 1,8-naphthimides. The results demonstrated that dendrimer **D3** and dendrimer **D4** completely inhibited the growth of Gram-positive bacteria after light treatment in solution, whereas only dendrimer **D4** had a similar effect on Gram-negative bacteria. Upon application to cotton fabrics, compound **D4** exhibited complete inactivation of Gram-positive bacterial growth upon light irradiation, suggesting its potential use as an antimicrobial photodynamic therapeutic agent for producing antimicrobial textiles. Moreover, given the ability of substance **D4** to produce at least two effects on viruses, it has been suggested that substances with similar structures may hold promise as inhibitors of herpes infections.

**Figure 16:**
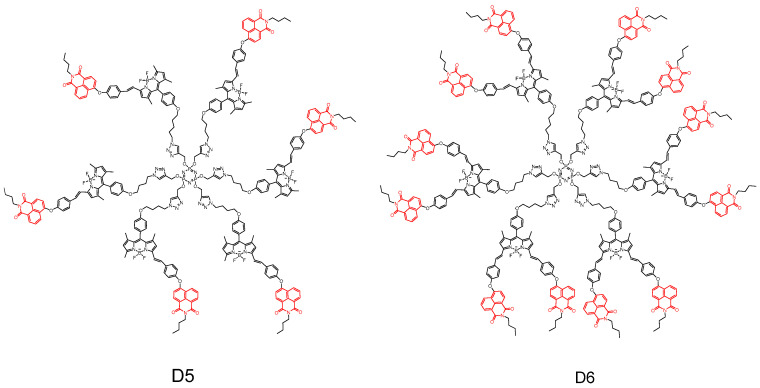
Chemical structures of naphthalimide–BODIPY–cyclotriphosphazene triads **D5** and **D6**.

In 2020, Şenkuytu et al. [54] reported a study on the synthesis and characterization of novel fluorescent naphthylimide (NI)–boronodipyridine (BODIPY) di-pair and dendritic triplex systems based on NI-functionalized mono- and diacyl BODIPY derivatives with cyclic triphosphononitrile core (Figure 16). The spectroscopic properties, including absorption, emission spectra, fluorescence quantum yield, and fluorescence lifetime of NI–BODIPY di-pair and NI–BODIPY–cyclotriphosphonitrile triplexes were investigated using UV-vis absorption and fluorescence emission (**D5** and **D6**) techniques. The presence of NI groups on the BODIPY core resulted in a red shift in the absorption and emission spectra compared to the BODIPY core alone. Furthermore, the quantum transfer process suppressed the emission of the NI groups, while inducing fluorescence from the BODIPY units. Additionally, both the dendrimer-based **D5** system and stilbene-based **D6** system exhibited strong absorption bands at approximately 570 nm under excitation by NI substituents, as well as at 639 nm under excitation by BODIPY substituents. Moreover, antimicrobial screening against Gram-positive *Staphylococcus aureus* revealed that the naphthylimide–BODIPY–cyclotriphosphonitrile triplex possessed significant antimicrobial activity.

#### 2.2.2. Naphthylimide-Dimer

**Figure 17:**
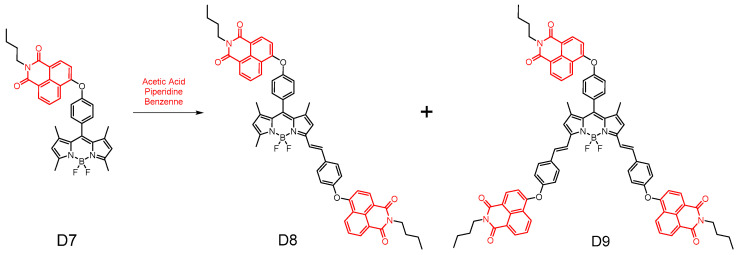
Pathway for synthesis of the Naphthalimide-BODIPY Dyads **D8 and D9** from **D7**.

In 2022, Hande E et al. [55] reported three novel meso-(**D7**), mono- and di-styryl-(**D8**), and di-styryl-(**D9**) naphthalimide–BODIPY binary compounds (Figure 17). Their spectroscopic properties, including steady state absorption, excited emission, and excited state spectra, were meticulously investigated for NI–BODIPY (**D7**) and NI–BODIPY–NI (**D8** and **D9**). Notably, all the resulting dibenzopyrrole compounds demonstrated robust absorption signals and moderate fluorescence characteristics. Furthermore, these NI–BODIPY derivatives demonstrated their potential utility in live cell imaging and intracellular staining applications. The antimicrobial studies revealed that these NI-fused BODIPY compounds exhibited a broad spectrum of activity against both Gram-positive cocci, such as *Staphylococcus aureus*, and Gram-negative bacilli, such as *Escherichia coli*. The conformational analysis elucidated that the NI moiety attached to the BODIPY influences the antibacterial potency. Among the series of compounds, **D9**, which harbors three NI units, emerged as the most active, effectively inhibiting the growth of both *E. coli* and *S. aureus,* with MIC values of 76 μM and 110 μM, respectively.

### 2.3. Anticancer Activity

Cancer currently ranks as the leading or second most prevalent cause of premature mortality in the majority of countries worldwide [56]. The development of novel anticancer medications with minimal toxicity, affordable cost, and high efficacy is crucial for improving the survival rate and quality of life for cancer patients [57,58,59]. DNA plays a crucial role in tumor growth and survival. Therefore, DNA has become one of the important biological targets for designing anticancer drugs [60,61,62].

#### 2.3.1. Amonafide Derivatives

Amonafide is a naphthylimide. Aminopyridine and its structural analogs disrupt the cleavage–release equilibrium of covalent adducts of DNA topoisomerase II, affecting its normal metabolism. Amonafide inhibits DNA synthesis, shows high DNA binding affinity and selectivity in clinical settings, and has demonstrated activity in stage II breast cancer trials. However, it is limited by dose-dependent myelotoxicity [63].

**Figure 18:**
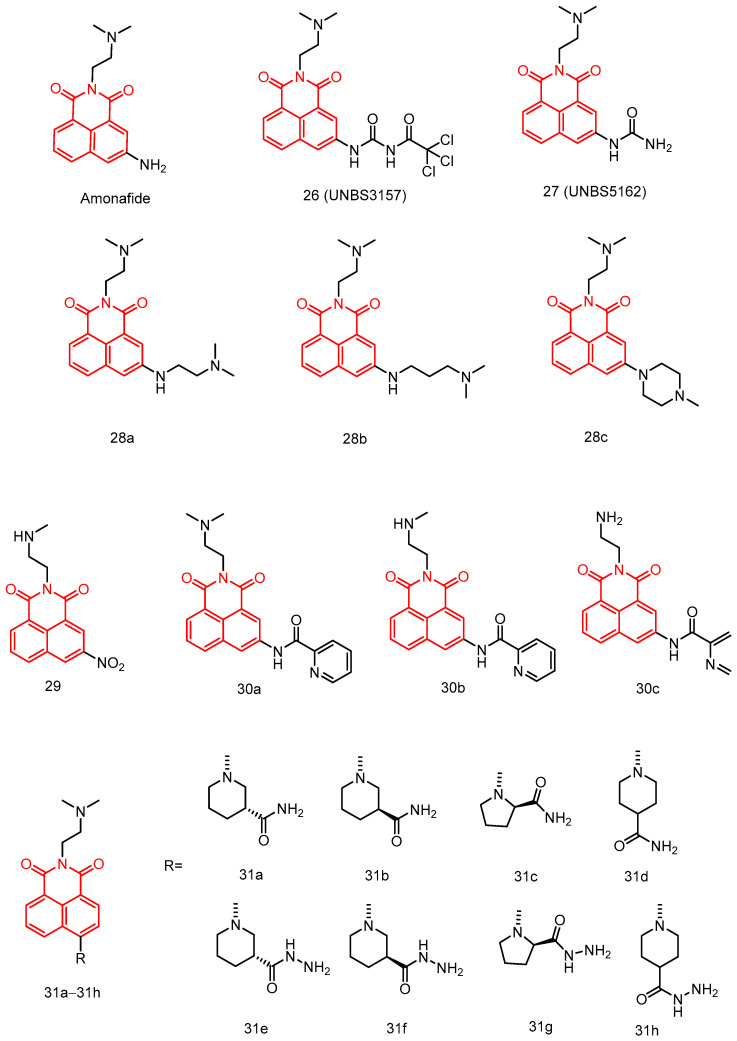
The structure of amonafide derivatives.

In 2007, Quaquebeke et al. [64] reported a novel anticancer naphthylimine with a unique mechanism of action, particularly inducing autophagy and senescence in cancer cells. Compound **26** UNBS3157 (Figure 18) has a maximum tolerated dose 3–4 times higher than aminopyralid and does not cause hematotoxicity in mice at doses that show significant antitumor effects. Additionally, compound **26** was shown to be superior to amonafide in vivo in the L1210 mouse leukemia model, the MXT-HI mouse mammary adenocarcinoma model, and the human A549 NSCLC and BxPC3 pancreatic carcinoma in situ models. Thus, compound **26** deserves further investigation as a potential anticancer agent.

In 2008, Mijatovic et al. [65] reported that UNBS3157 could be rapidly and irreversibly hydrolyzed to compound **27** UNBS5162 (Figure 18) without producing aminyl. After repeated administration, UNBS5162 significantly improved survival in an in situ human prostate cancer model. The two novel naphthylimides, UNBS3157 and UNBS5162, have unique mechanisms of action. UNBS5162 is a pan-antagonist of CXCL chemokine expression and showed antitumor effects when administered alone in an experimental model of refractory human prostate cancer. Additionally, it enhanced taxol activity when co-administered with taxol.

In 2008, Xie et al. [66] reported a series of amonafide analogs showing potential anticancer activity against HeLa and P388D1 cell lines see Table 2 below, with **28a**, **28b**, and **28c** (Figure 18) showing superior activity to amonafide in HeLa cells. DNA binding studies showed that they bind DNA through a similar amonafide pattern. More importantly, the new analogs can avoid the side effects of amonafide, due to their structure lacking a primary amine at 5-position.

In 2020, Xin et al. [67] reported a series of 3-nitronaphthalimides as antitumor agents. These compounds showed significant inhibitory activities against SKOV3, HepG2, A549, T-24, and SMMC-7721 cancer cells see Table 2 below. Among them, compound **29** (Figure 18) exhibited the strongest inhibitory activity against HepG2 and T-24 cells compared to mitonafide, with IC_50_ values of 9.2 ± 1.8 μM and 4.133 ± 0.9 μM, respectively. Mechanistic studies indicated that compound **1a** induced DNA damage and topoisomerase I inhibition in T-24 cancer cells. It caused G2 phase arrest and apoptosis by upregulating the expression levels of cyclin B1, cdc2-pTyr, Wee1, γ-H2AX, p21, Bax, and cytochrome c, and downregulating Bcl-2 expression.

In 2023, Ge et al. [68] reported several newly prepared naphthylimide derivatives and their biological properties. Some of these derivatives demonstrated notable activity see Table 2 below. Among them, compounds **30a**, **30b**, and **30c** (Figure 18) showed the highest activity against A549 cells, with IC_50_ values around 3 μM. The anticancer activity could be fine-tuned by modifying the 3-position of the naphthylimide ring and side chain. The activity followed the order: -NO_2_ ≈ pyridine and tertiary nitrogen atoms ≈ secondary nitrogen > primary nitrogen. Compounds **30a** and **30b** significantly damaged DNA. Furthermore, compounds **30a** and **30b** induced significant autophagy in A549 cancer cells. Both compounds possessed the ability to inhibit FTO demethylase activity, which could explain their high antiproliferative capacity. These potentially useful naphthylimides could facilitate research on cancer therapeutics, including the mechanisms of the autophagic death pathway and inhibition of FTO demethylation.

In 2021, Wang et al. [69] reported a series of naphthimide derivatives affixed with amide/acylhydrazide-modified piperidine and pyrrolidine derivatives (Figure 18). The in vitro antiproliferative activity was evaluated against HeLa, HepG2, and A549 tumor cell lines see Table 2 below. The cytotoxicity of amide-modified naphthimide derivatives **31a** and **31c** was superior to that of acylhydrazine-modified **31e** and **31g** against HeLa and HepG2 cells. Derivative **31a** showed IC_50_ values of 5.59 μM and 5.06 μM, respectively. Acylhydrazine-modified **31f** and **31h** showed better cytotoxicity than amide-modified **31b** and **31d**. The IC_50_ values of **31f** against HeLa and HepG2 cells were 5.79 and 4.89 μM, respectively. The chiral conformations in the **31a** and **31f** piperidine groups had an opposite effect on the cytotoxic activity, which was superior to that of the control drug, aminoglycoside. These results suggest that the chiral configuration and substituents are crucial for cytotoxic activity.

#### 2.3.2. Alkylated Naphthylimide Analogues

**Figure 19:**
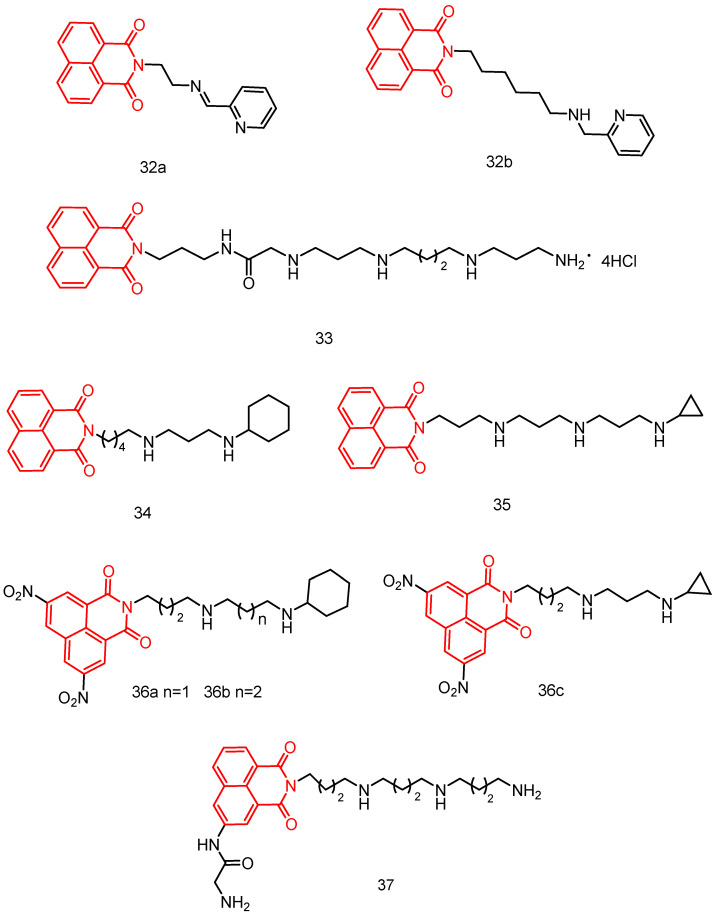
The structures of compounds **32**–**37**.

In 2013, Seliga et al. [70] reported a series of naphthylimine polyamine couplers (Figure 19) and evaluated their in vitro antiproliferative activity against human leukemia (Jurkat), human cervical adenocarcinoma (HeLa), human mammary adenocarcinoma (MCF-7), and human lung adenocarcinoma (A549) cell linessee Table 2 below. Among the six derivatives, the new **32a** and **32b** had the highest antiproliferative activity, with IC_50_ values ranging from 5.67 to 11.02 µM. Additionally, fluorescence spectroscopy revealed that the **32b** compound displaced the intercalating agent ethidium bromide from calf thymus DNA. The apparent binding constant was estimated to be 3.1 × 10^6^ M^−1^, indicating a non-insertion mode of DNA binding. These findings suggest that naphthylimine polyamine splices can rapidly penetrate cancer cells.

In 2016, Li et al. [71] reported three aromatic imine scaffolds (Figure 19) coupled with different amine/polyamine sequences and evaluated their in vitro and in vivo antitumor activities see Table 2 below. The results showed that the 1,8-naphthimide and spermine concatenated **33** inhibited tumor cell proliferation and induced apoptosis via the ros-mediated mitochondrial pathway. In vivo experiments in three H22 tumor transplantation models demonstrated that compound **33** was effective in preventing lung cancer metastasis and prolonging life span. In addition, compound **33** inhibited tumor growth and improved body mass index more than the positive control aminonitramine.

In 2018, Dai et al. [72] reported a series of alkylation-modified naphthylimine-polyamine concaten (Figure 19). Among these, the cyclohexyl terminally modified naphthylimine-polyamine concatenate **34** exhibited superior cytotoxicity (IC_50_ = 0.14–10.94 μM) compared to other new concatenates see Table 2 below. Compound **34** triggered ROS generation via a p53-mediated pathway, disrupting the mitochondrial membrane potential and inducing intrinsic cellular apoptosis and migration inhibition.

In 2018, Li et al. [73] reported a novel class of polyamine analogs with alkylation modifications at the end of the polyamine chain (Figure 19). Among these, compound **35** showed promising activity against a wide range of cancer cells (IC_50_ = 0.87–6.34 μM) see Table 2 below, induced cellular autophagy via ROS generation, and significantly inhibited migration by downregulating the expression of MMP9 and *β*-catenin.

In 2020, Ma et al. [74] reported a new class of polyamine-based naphthylimide concatenates **36a**–**c** (Figure 19), most of which exhibited good activity (IC_50_ = 0.08–3.62 μM) see Table 2 below. Among these, the dinitronaphthylimide derivative **36c**, with a 4,3-cyclopropyl group sequence, preferentially accumulated in cancer cells both in vitro and in vivo, showing minimal side effects compared to aminamide. Importantly, the in vivo antitumor effect of **36c** at a low dose of 3 mg/kg (57.97%) was superior to that of the positive control at 5 mg/kg (53.27%). A higher dose of 5 mg/kg (65.90%) resulted in significantly increased antitumor activity and reduced toxicity. Upregulation of p53 and an increase in apoptotic cells (73.50%) suggested that **36c**-induced apoptosis might be related to enhanced DNA damage. Besides targeting DNA, **36c** regulates polyamine homeostasis by upregulating polyamine oxidase (PAO) in a manner different from aminopyrimidine. By targeting PTs, overexpressed in most cancer cells, **36c** downregulates Put, Spd, and Spm, inhibiting rapidly growing tumor cells. The study demonstrated that naphthylimide coupling exhibits significant activity and low-dose toxicity in the treatment of hepatocellular carcinoma.

In 2021, Ma et al. [75] reported a novel class of naphthylamine–polyamine couplers (Figure 19). Among them, polyamine coupler **37** demonstrated more potent antitumor activity against hepatocellular carcinoma cell lines, including HepG-2 and Huh-7, compared to other compounds and cell lines. It was also significantly more potent than amonafide and cisplatin, with potencies in the nanomolar range. The in vivo antitumor and antimetastatic effects (76.01% and 75.02%, respectively) were significantly higher than those of the positive control amonafide (46.91% and 55.77%, respectively) at a dose of 5 mg/kg. Potential molecular mechanisms suggest that compound **37** regulates polyamine metabolism and function by targeting lysosomes in a manner completely different from amonafide. Additionally, it induces DNA damage through the upregulation of p53 and γH2AX.

#### 2.3.3. Study on Bis-Naphthalimide Derivatives

**Figure 20:**
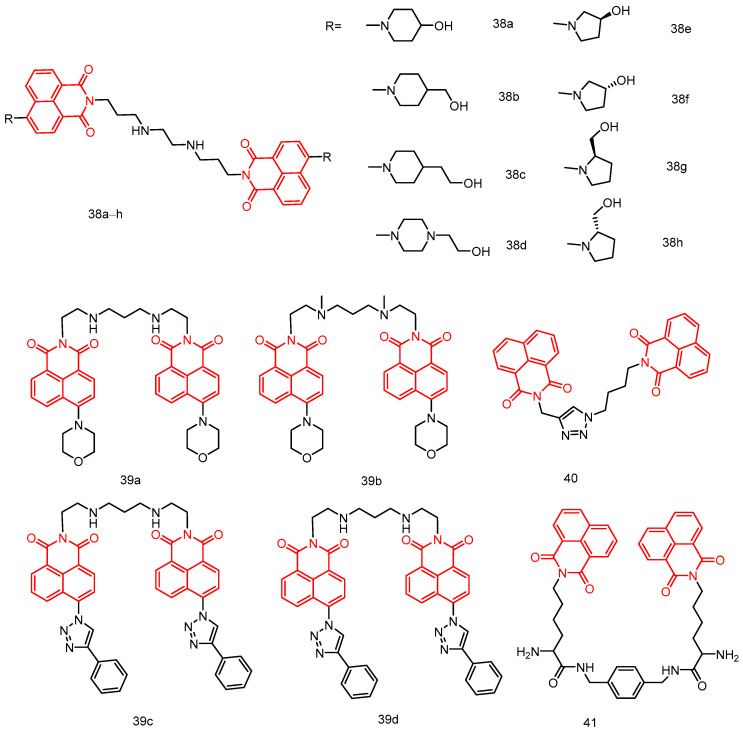
The structure of bis-naphthalimide derivatives **38**–**41**.

In 2018, Rong et al. [76] reported a series of novel saturated nitrogen heterocyclic *N*, *N*-bis(3-aminopropyl)methanamine-bridged bis-naphthylimine derivatives **38a**–**h** (Figure 20), all the compounds exhibited certain cytotoxic activities against Hela, MCF-7, A549, and MGC-803 cells see Table 2 below, among which compounds **38a** and **38d** showed strong cytotoxic activities against Hela cells and MGC-803 cells, with IC_50_ values of 2.89, 0.60, 2.73, and 1.60 µM, respectively, which were better than that of the control drug amonafide. Mechanistic studies showed that compound **38a** had a stronger intercalation with DNA, while compound **38d** had a weaker intercalation with DNA, and due to additional electrostatic interactions, compound **38d** had a stronger binding effect with DNA stronger. In addition, the lysosomal targeting behavior of compounds **38a** and **38d** and their imaging ability were investigated through colocalization experiments, and it was found that **38a** and **38d** had a fluorescence imaging function with Hela cells in lysosomes.

In 2019, Ou et al. [77] reported four bis-naphthalimide derivatives **39a**–**d** (Figure 20) with polyamine linkages, and compounds **39a**, **39b,** and **39d** showed high affinity for telomeric and oncogenic G-quadruplexes. Compound **39c** only showed high affinity (Ka > 106 M^−1^) for c-kit and telomeric quadruplexes, and the interaction with c-myc quadruplexes and double-stranded CT DNA was weaker. These compounds effectively inhibited the growth of A549 cancer cell line with low cytotoxicity against non-cancerous MRC-5 cells, with IC_50_ values in the range of 0.15 to 1.04 µM see Table 2 below.

In 2019, Shankaraiah et al. [78] reported a new series of 1,2,3-triazole–naphthimide couplers. Among these, compound **40** (Figure 20) exhibited significant cytotoxic activity against A549 lung cancer cells, with an IC_50_ of 7.6 ± 0.78 µM. Relative viscosity measurements and molecular docking studies indicated that compound **40** binds to DNA via intercalation.

In 2018, Huang et al. [79] reported a series of dinaphthylimide derivatives with different diamine linkers, and SAR analyses showed that the length of the diamine linker had a significant effect on the binding ability of the dinaphthylimide derivatives. Compounds with shorter linkers have larger binding constants and classical binding modes. Compound **41** (Figure 20) with a rigid p-dimethylenediamine linker showed better cytotoxicity than the other dinaphthylimide derivatives.

#### 2.3.4. *N*-Substituted 1,8-Naphthalimide Derivatives

**Figure 21:**
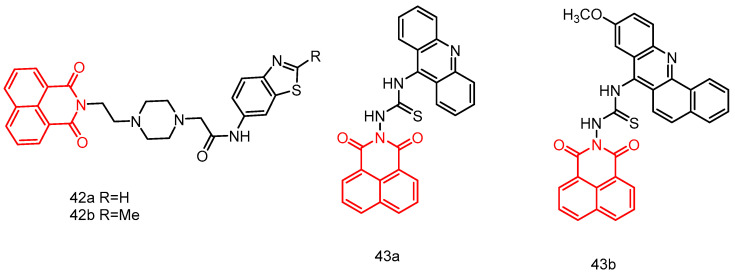
The structures of compounds **42**–**43**.

In 2018, Rao et al. [80] reported a series of naphthylimide-benzothiazole derivatives. Derivatives **42a** and **42b** (Figure 21), obtaining a 6-aminobenzothiazole ring, exhibited cytotoxicity against colon cancer cells (IC_50_ of 3.715 and 3.467 μM, respectively) and lung cancer cells (IC_50_ of 4.074 and 3.890 μM, respectively). The possible mechanism of their antitumor effects is the inhibition of DNA topoisomerase II.

In 2020, Chen et al. [81] reported two 1,8-naphthimide-acridinylthiourea hybrids, **43a** and **43b** (Figure 21), which exhibited improved anticancer activity (IC_50_ = 14.66 ± 0.31 μM) against six human tumor cell lines, especially the MT-4 cell line. Compound **43b** is considered a potential Topo I inhibitor with superior anticancer activity. Compound **43a** demonstrated significantly better AChE inhibitory activity than **43b**. Compounds **43a** and **43b** warrant further investigation as representative Topo I or AChE inhibitors.

#### 2.3.5. 1,8-Naphthalimide Derivatives

**Figure 22:**
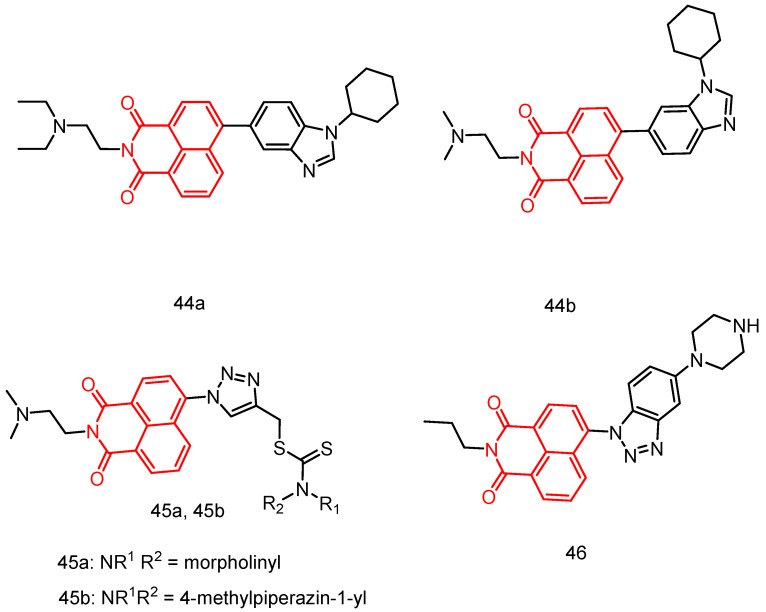
The structure of compounds **44**–**46**.

In 2019, Singh et al. [82] reported a series of substituted naphthylimine benzimidazole isomers tested against 60 human tumor cell lines. Most of the compounds showed good activity. Notably, compounds **44a** and **44b** (Figure 22) exhibited higher activity against leukemia and colon cancer subgroups, with MG_MID GI50 values of 1.43 and 1.83 μM, respectively. These compounds may bind to ct-DNA through an embedding mode, resulting in strong biological activity. Additionally, the transport behavior suggested that these molecules can efficiently bind and be carried by bovine albumin, with hydrogen bonding and hydrophobic interactions playing important roles in their interaction with serum albumin.

In 2021, Chen et al. [26] reported 15 novel dithiocarbamate-derived naphthoimides. Among these, compound **45a** (Figure 22), containing a morpholinyl substituent, showed the highest activity and selectivity against HepG-2 cancer cells, with an IC_50_ of 10.86 µM. The most active compound **45b**, exhibited an IC_50_ value of 2.27 µM against A549 cells, which is comparable to the level of cisplatin. The introduction of a triazole ring enhanced the activity of naphthoimides. Additionally, some naphthoimides with 4-position substituents exhibited higher activity.

In 2022, Wang et al. [83] reported nine novel naphthylimide-benzotriazole concatenates. Among these, compound **46** (Figure 22) exhibited the best antitumor activity in A549 cells, with an IC_50_ value of 6.73 ± 0.37 μM. Compound **46** selectively interacted with the G-quadruplex DNA in the BCL2 promoter region, significantly influencing the expression of the BCL2 gene in A549 cells. Further experiments confirmed that these effects of compound **46** led to apoptosis, DNA damage, and induction of autophagy in A549 cells.

**Figure 23:**
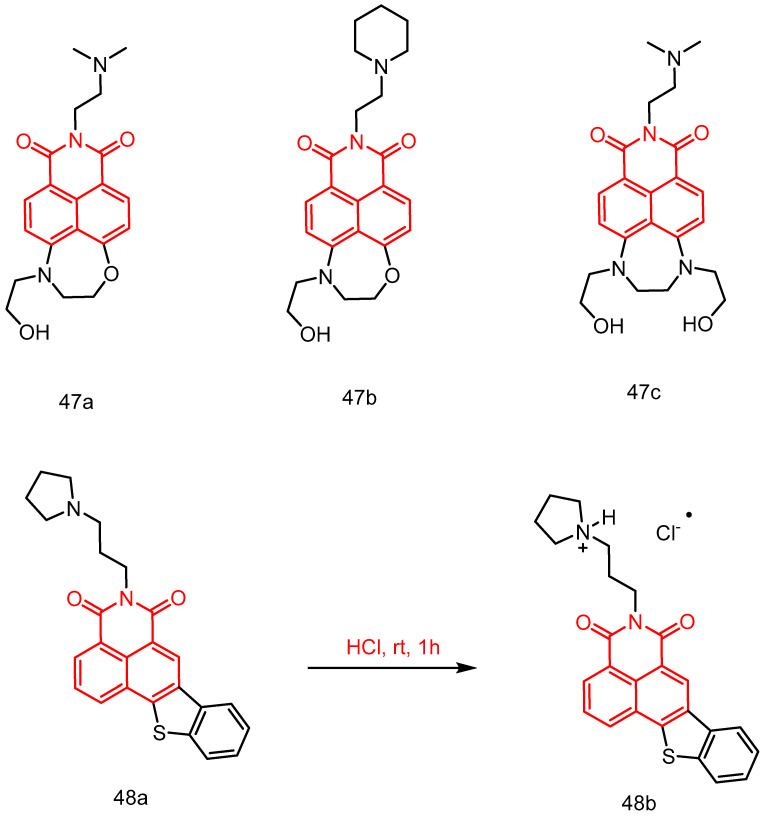
The structures of compounds **47**–**48**.

In 2022, Yang et al. [49] reported a series of seven-membered cyclic naphthimide derivatives. Their inhibitory effects on the growth of A549 and HL60 tumor cells were evaluated see Table 2 below, and their structure–activity relationships were summarized. Among them, *N*′,*N*′-dimethylethane-1,2-diamine derivatives **47a** and **47c** (Figure 23), and their *N*-(2-aminoethyl)pyrrolidine analogues **47b,** exhibited good antiproliferative activity (IC_50_ = 0.9–5.5 μmol/mL) in A549 and HL60 cells. They were also effective in inhibiting the growth of human esophageal, colon, and breast cancer cells in the single-digit micromolar range, similarly to amonafide. The photophysical properties of **47a**–**c** were also tested. The asymmetric derivatives (**47a** and **47b**) showed a high fluorescence quantum yield in water (Φ = 0.47), a rare property among naphthylimide derivatives.

In 2022, Huang et al. [84] reported a series of new naphthoimide derivatives, specifically benzothiophene naphthoimides. Among these, compound **48a** (Figure 23) showed the strongest antitumor activity, with an IC_50_ value of 0.59 ± 0.08 μM and the best selectivity for HepG2 cells. Compound **48b** is the hydrochloride salt of the corresponding compound **48a**. Compound **48a** selectively induced the formation of a G-tetrameric structure (G4) from the G-rich HRCC DNA sequence in mitochondria and stabilized it. This mediated a decrease in the mitochondrial membrane potential and the generation of reactive oxygen species, leading to mitochondrial dysfunction. Finally, by promoting the expression of p-Erk1/2, it led to proliferation inhibition, apoptosis, and protective autophagy in cancer cells. In addition, compound **48b**, as a salt of **48a**, exhibited significant in vivo antitumor effects in a HepG2-xenograft mouse model, showing 51.4% tumor growth inhibition at a dose of 15 mg/kg.

**Figure 24:**
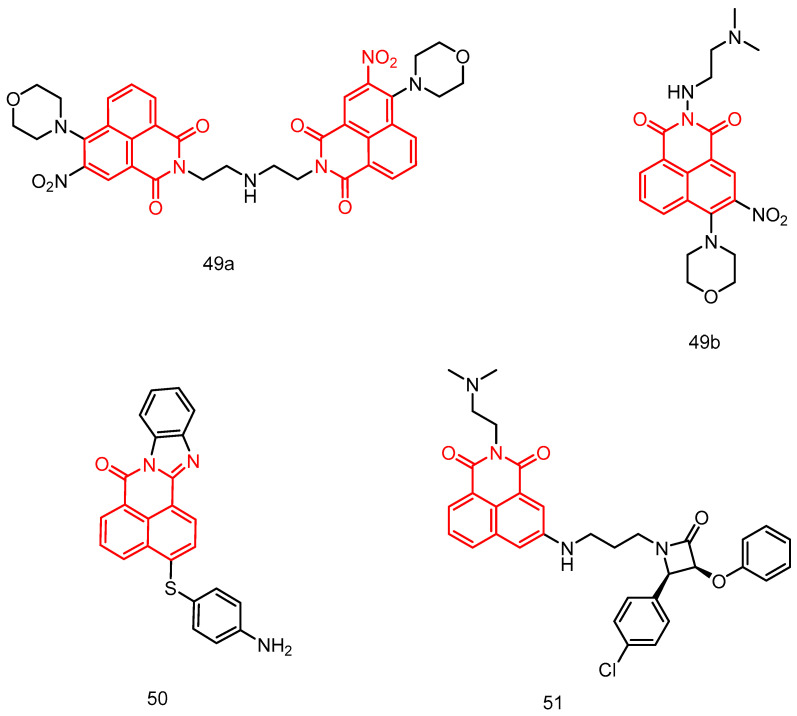
The structure of compounds **49**–**51**.

In 2023, Chen et al. [85] reported a series of mono- and di-naphthoimide derivatives containing 3-nitro, evaluating their in vitro anticancer activity see Table 2 below. Some of these compounds exhibited better antiproliferative activity on cell lines compared to mitonafide and aminopyridine. Notably, dinaphthylimide **49a** (Figure 24) was identified as the most potent compound against mfc-803 cell proliferation, with an IC_50_ of 0.09 μM, significantly lower than mononaphthylimide **49b**, mitonafide, and aminopyridine. Gel electrophoresis analysis revealed that DNA and Topo I were potential targets of compounds **49a** and **49b**. Treatment of CNE-2 cells with compounds **49a** and **49b** resulted in S-phase cell cycle blockade, upregulation of the anticancer gene p27, and downregulation of CDK2 and cyclin E expression levels. These results indicate that the cells expressed the anticancer gene p27 during the S-phase.

In 2013, Verma et al. [86] reported a series of amine-substituted naphthylamine analogs and evaluated their in vitro antitumor activity against 60 tumor cell lines at a single dose concentration of 10 mM. These analogs showed potential antitumor activity against a wide range of tumor cell lines. Compound **50** (Figure 24) was five times more active than the standard antitumor drug 5-fluorouracil (5-FU), with a GI50 and TGI of MG-MID of 5.05 and 38.71, respectively. The ct-DNA binding studies of most of the active **50** compounds showed strong interaction properties. Moreover compound **50** has good physical properties, with good pharmacokinetics and drug bioavailability.

In 2019, Rad et al. [87] reported a potent DNA embedding agent, *β*-lactam compound **51** (Figure 24), which is potentially cytotoxic to HepG2 cancer cells, with an IC_50_ of 65.5 μM and 34.2 μM after 48 h and 72 h of incubation, respectively. Gel electrophoresis confirmed its insertion into pBluescript plasmid DNA, resulting in a significant decrease in its electrophoretic mobility. Gel electrophoretic profiles showed that *β*-lactams were tightly stacked between DNA bases via the major groove, and molecular docking experiments also showed the major groove binding behavior of *β*-lactams.

#### 2.3.6. Naphthalimide Metal Complexes

**Figure 25:**
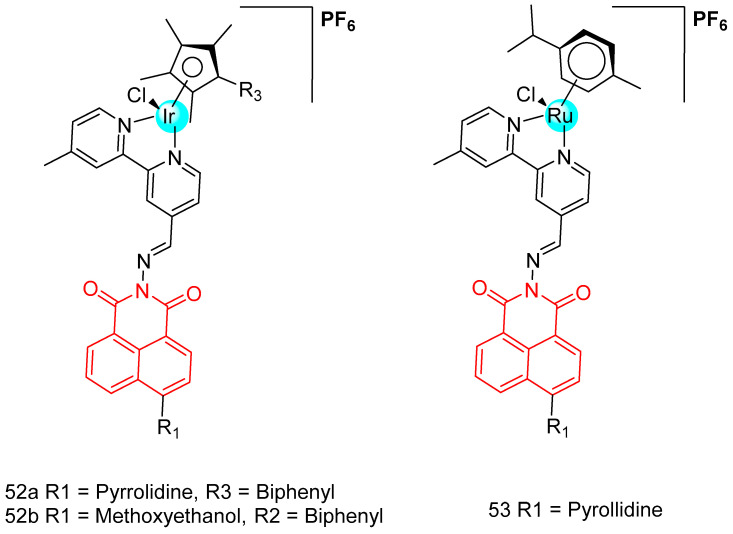
The structures of 1,8-naphthimide half-sandwiched iridium(III) and ruthenium(II) complexes.

In 2019, Ma et al. [88] reported five naphthylimide-modified semi-sandwiched iridium-ruthenium complexes (Figure 25) and investigated the anticancer activity of the complexes against a wide range of cancer cells. Among them, complexes **52a** and **52b** showed better anticancer activity than cisplatin, iridium complex **52a** showed the highest activity with IC_50_ = 11.30–15.60 µM, and changing the metal ions to metallic ruthenium **53** resulted in loss of activity against all cancer cells (IC_50_ > 50 µM). By changing the ligands, the complexes exhibited different photophysical properties and their mechanism of action to enter the cells and induce apoptosis was also different. Complex **52a** successfully targeted mitochondria and complex **52b** targeted lysosomes, causing mitochondrial damage and lysosomal damage and inducing apoptosis. Excitingly, complex **52a** had good anti-metastatic ability against cancer cells. Complexes **52a** and **52b** had no significant effect on the NADH binding response, but had a moderate binding capacity to bovine serum albumin.

**Figure 26:**
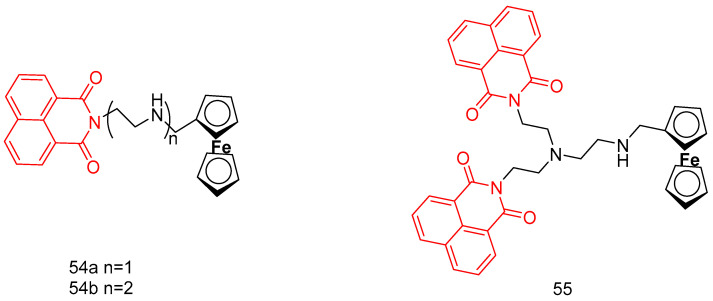
The structures of ferrocene-attached 1,8-naphthimide derivatives.

In 2019, Jia et al. [89] reported three novel ferrocene-attached naphthylimine derivatives (**54a**, **54b**, and **55**) (Figure 26), and the hybrid compound **55** was 6.45~17.62 times more toxic to tumor cells than the control drug aminopyrimidine. The synergistic effect of the ferrocene moiety played an important role in enhancing the cytotoxicity of the dinaphthylimide derivatives. The ferrocene derivatives (**54a**, IC_50_ > 200 µM and **54b**, IC_50_ = 68.54–113.7 µM) were less active than aminocarbamoyl (IC_50_ = 34.64–129 µM) due to the lack of protonated amino substituents. EB display, UV-visibility, and viscosity studies showed that ferrocene-attached naphthanilide derivatives have a partial intercalation binding mode. The ferrocene moiety contributes to the DNA binding ability of the dinaphthylimide derivatives. The cytotoxicity of compound **55** was associated with DNA damage in tumor cells.

**Figure 27:**
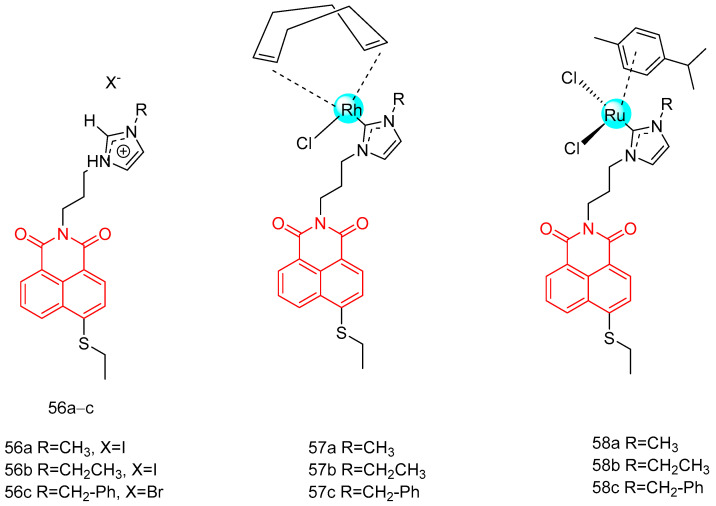
The structures of rhodium(I) and ruthenium(II) complexes of 1,8-naphthimides.

In 2018, Streciwilk et al. [90] reported fluorescent 4-ethylthio-1,8-naphthalimides containing rhodium(I) N-heterocyclic carbene (NHC) and ruthenium(II) NHC fragments (Figure 27). They evaluated these compounds for their antiproliferative effects and DNA binding activity. The metal-free derivatives (**56a**–**c**) showed good activity against MCF-7 cells (IC_50_ = 1.50–46.00 µM) and moderate activity against HT-29 cells (IC_50_ = 9.60–27.50 µM). Increased lipophilicity of the alkyl group at the three positions on the imidazole ring enhanced the activity in the order: methyl (**56a**) > ethyl (**56b**) > benzyl (**56c**). Rhodium complexes were more active than ruthenium complexes: Rhodium (**58a-c**, IC_50_ = 4.80–18.60 µM, MCF-7 IC_50_ = 1.70–5.80 µM, HT-29 IC_50_ = 6.20–10.00 µM) and Ruthenium (**57a-c**, IC_50_ = 1.70–5.80 µM, MCF-7 IC_50_ = 4.80–18.60 µM, HT-29 IC_50_ = 4.90–36.80 µM). Both organometallic types triggered ligand-dependent potent cytotoxic effects on tumor cells, with rhodium(I) NHC derivatives producing stronger effects than ruthenium(II) NHC analogues. Intensive studies on the naphthylimide interaction with DNA confirmed that naphthylimide fragments intercalate between planar bases of B-DNA and stack on top of the quaternary g-tetrameric structure. This intercalation mechanism confers potent pharmacological effects.

**Figure 28:**
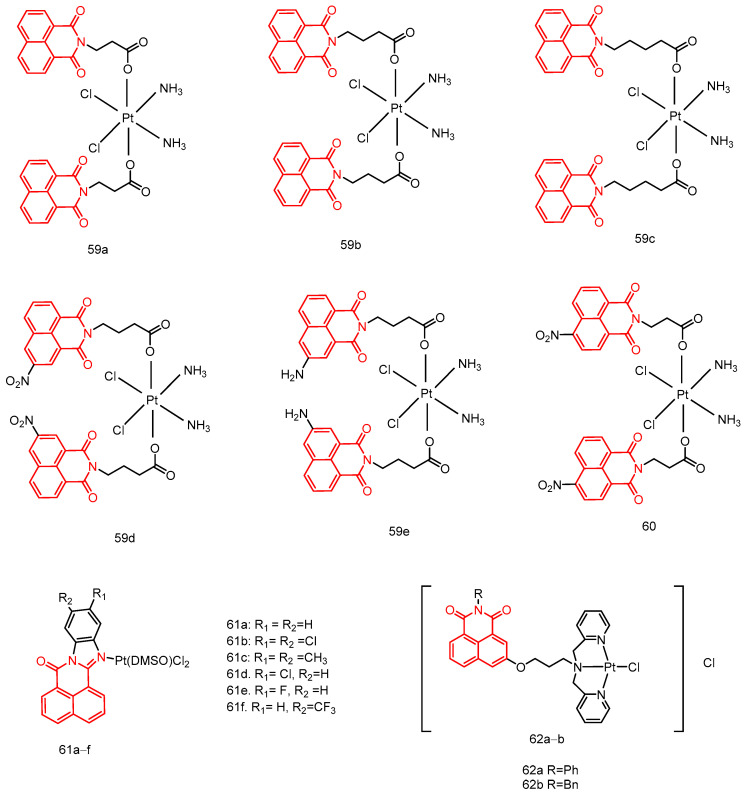
The structures of platinum(IV) complexes with 1,8-naphthimide.

In 2018, Wang et al. [91] reported a series of naphthylimide platinum(IV) compounds (**59a**–**e**) (Figure 28) with a dual DNA damage mechanism. Some of the naphthylimide platinum(IV) compounds have significant antitumor activity see Table 2 below, especially compounds **59b** and **59e**, whose antitumor activity was comparable to or even superior to that of the positive controls cisplatin and oxaliplatin, and which has great potential for overcoming platinum(II) drug resistance. Platinum(IV) compounds bind to DNA and cause damage to DNA via naphthylimide fragments. Platinum(II) complexes released in a reducing microenvironment cause significant secondary DNA damage. Naphthylimine platinum(IV) complexes can bind efficiently to HSA via electrostatic forces, thereby affecting drug distribution and biological activity in vivo. In addition, the accumulation of the tested platinum(IV) compounds in whole cells and DNA was significantly enhanced compared to cisplatin and oxaliplatin.

In 2020, Li et al. [92] reported a mononitronaphthylimide Pt(IV) complex **60**, which exhibited few side effects (Figure 28). Complex **60** targeted the DNA damage response to overcome cisplatin resistance via a dual DNA damage approach. It showed significant antitumor activity in vivo (70.10%) compared to cisplatin (52.88%), with the highest ploidy increase (FI) in A549cisR cells (5.08) and the lowest FI (0.72) in A549 cells, indicating preferential accumulation in drug-resistant cell lines. The molecular mechanisms suggest that complex **60** targets drug-resistant cells differently than existing Pt drugs. It promotes p53 gene and protein expression more efficiently than cisplatin, resulting in enhanced anticancer activity. The up-regulation of γ-H2AX and downregulation of RAD51 suggest that complex **60** induces severe DNA damage and inhibits DNA repair, making it more cytotoxic than cisplatin. The preferential accumulation of complex **60** in cancer cells (SMMC-7721) compared to normal cells (HL-7702) suggests a favorable safety profile for clinical treatment. Additionally, the high therapeutic index of complex **60** in 4T1 cells in vivo highlights its potential in breast cancer therapy.

In 2019, Liang et al. [93] reported a series of naphthylbenzimidazole–platinum complexes (**61a**–**f**) (Figure 28) as antitumor agents. In vitro studies showed that these complexes exhibited medium-high antiproliferative activity against various cancer cells see Table 2 below, with significant sensitivity and selectivity for SMMC-7721 and U251 cell lines, and low toxicity to normal HL-7702 cells. In vivo assays revealed that complexes **61a** and **61e** demonstrated significant antiproliferative activity compared to cisplatin in NCI-460 and SMMC-7721 models, respectively. Complexes **61a** and **61e** showed superior activity to cisplatin against A549CDDP and SKOV3CDDP cell lines, with IC_50_ values of 6.98 ± 0.47 µM, 5.62 ± 0.88 µM, 13.13 ± 2.11 µM, and 5.30 ± 0.33 µM, respectively. Against A549 and SKOV3 cell lines, the IC50 values were 7.32 ± 0.51 µM, 5.19 ± 0.49 µM, 14.92 ± 0.11 µM, and 12.19 ± 0.92 µM, respectively. These results suggest that incorporating naphthylbenzimidazole in platinum complexes may overcome drug resistance. Mechanistic studies indicated that complexes **61a** and **61e** primarily exert their antitumor effects through covalent DNA binding and upregulation of intracellular topo I levels, differing from the mechanism of cisplatin.

In 2019 Huang et al. [94] reported two novel platinum(II) complexes with naphthylimine derivatives as ligands (Figure 28), in which **62b**-Pt showed higher in vitro antitumor activity (0.89 ± 0.25 μM) than **62a**-Pt (11.32 ± 0.47 μM) and cisplatin (11.09 ± 1.01 μM) against human non-small cell lung cancer NCI-H460 cells. In addition, **62b**-Pt induced apoptosis in NCI-H460 cells by inhibiting telomerase activity and mitochondrial dysfunction.

### 2.4. Other Biological Activities of Naphthalimides (As Antimalarial, Antiviral, Anti-Inflammatory, Antithrombotic, and Antiprotozoal Agents)

In addition to being widely used for their antibacterial and anticancer activities, naphthylimide derivatives also show other pharmacological characteristics. Some of the important studies are summarized below.

**Figure 29:**
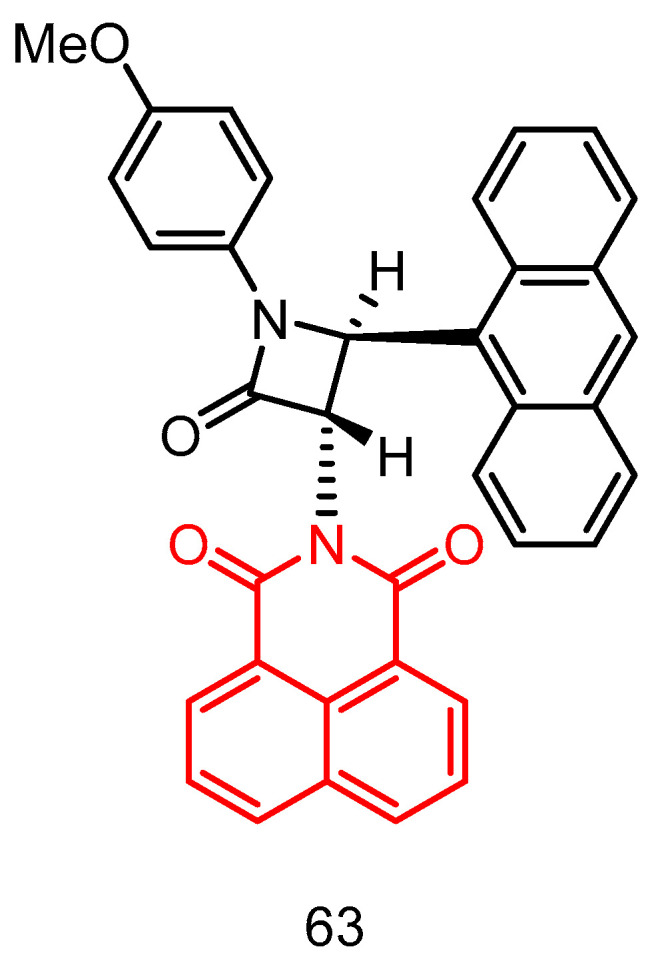
Chemical structure of compound **63**.

In 2017, Rad et al. [95] reported a series of naphthylimine-substituted *trans*-*β*-lactams using a stereoselective enone-imine cycloaddition reaction (Staudinger reaction). To the best of our knowledge, this was the first time that an azetidinone had been used for the synthesis of a 2-azadione. This novel enone exhibited excellent yields and unique trans diastereoselectivity. Furthermore, we were able to demonstrate that these derivatives were effective as antimalarials against chloroquine-resistant strains, with an IC_50_ of 3 µM for compound **63** (Figure 29). Further studies were focused on the synthesis of other analogs to enhance the antimalarial Plasmodium bioactivity and drug-like properties of the *β*-lactams and to better understand the basis of the observed antimalarial properties.

**Figure 30:**
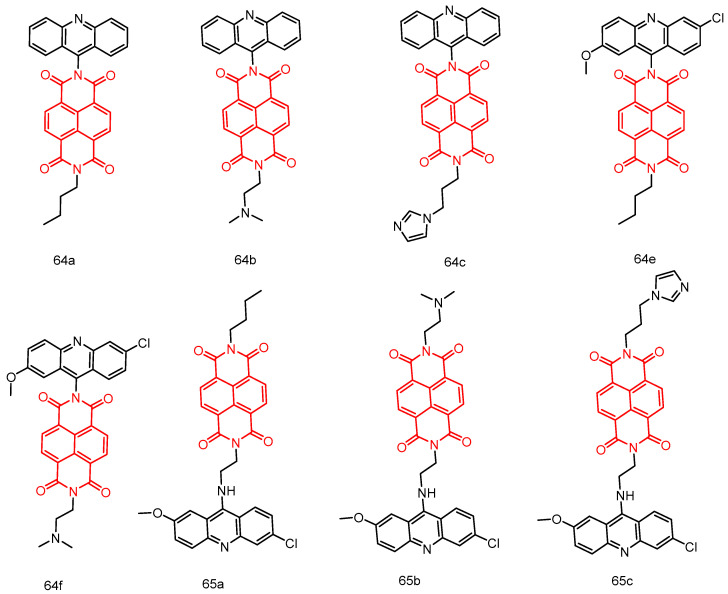
Chemical structures of the compounds **64a**–**f**, **65a**–**c**.

In 2016, Dana et al. [96] reported a new class of bifunctional molecules by directly combining acridine (Ac) and redox-active naphthalenediimide (NDI) via a flexible linker (en) (Figure 30). Evaluation of the in vitro activity of these couplers against Pf 3D7 and Pf W2 strains showed that the orthogonal Ac-NDI series (**64a**–**e**) were active in the micromolar to submicromolar range, and the flexible Ac-en-NDI series (**65a**–**c**) were active in the nanomolar range. Among the flexible Ac-en-NDI molecules, the IC_50_ values of **65a** and **65b** against Pf 3D7 were 3.65 and 4.33 nM, respectively, against Pf W2 strain, the IC_50_ values of **65a** and **65b** were 52.20 and 28.53 nM, respectively, which were about one order of magnitude higher than those of the standard drug CQ. SAR studies showed that the aminoethyl spacer between the Ac and NDI pharmacophores is essential for exhibiting potent antiplasmodial activity. Evaluation of theoretical physicochemical properties showed that conjugates **65a** and **65b** almost conformed to Lipinski’s rule of five nulls. In addition, these potent conjugates were found to have low cytotoxicity against mammalian cell lines.

**Figure 31:**
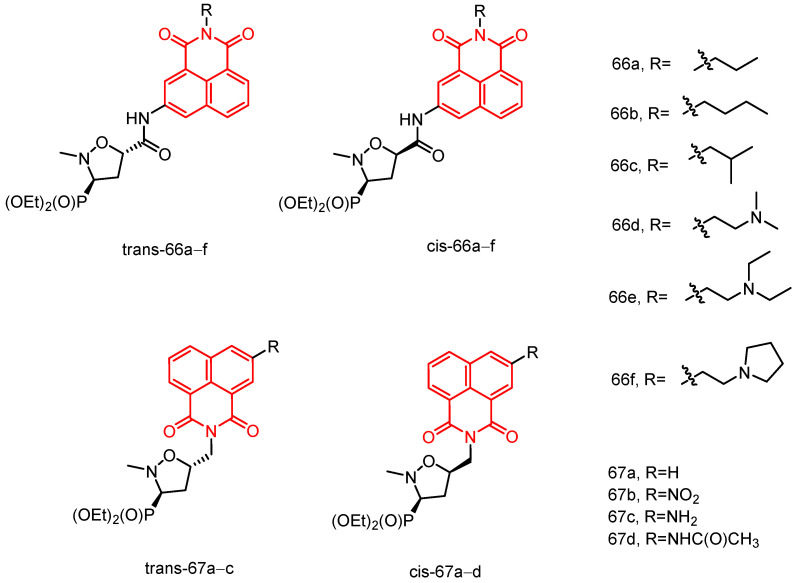
Chemical structures of the compounds **66a**–**f**, **67a**–**d**.

In 2015, Kokosza et al. [97] reported a new series of 5-arylcarbamoyl and 5-arylmethyl-2-methylisoxazolidine-3-acyl-3-phosphonates (Figure 31). All the *cis*- and *trans*-isoxazolidine phosphonates obtained were evaluated for their antiviral activity against a wide range of DNA and RNA viruses. Some of these derivatives showed activity against varicella-zoster virus and cytomegalovirus. Of all the compounds tested, isoxazolidines *trans* **66d** and *trans* **66f** showed the highest activity against cytomegalovirus (EC_50_ = 8.9 µM), which was comparable to the activity of the approved drug acyclovir. Isoxazolidine compounds (except *trans* **66c**) were active against TK VZV strains, and compound *cis*-**66d** showed better potency (EC_50_ = 20 µM) than the reference drug acyclovir (EC_50_ = 33–44 µM), which is also an approved drug. Some of the compounds also showed antiviral activity against HSV and poxviruses (*cis* and *trans* **66d**, *cis* and *trans* **66f**, EC_50_ = 45–58 µM), Coxsackie B4 and Punta Toro viruses (**67a** and **67d**, EC_50_ = 45–73 µM). Although compounds 67a and 67d inhibited Coxsackie B4 virus with better activity than the reference compound ribavirin, the reference compound, they were not potent enough to warrant further investigation. In general, *trans*-**66**/*cis*-**66** of compounds containing a carbamoyl linker are more active than *trans*-**67**/*cis*-**67** containing a methylene bridge.

**Figure 32:**
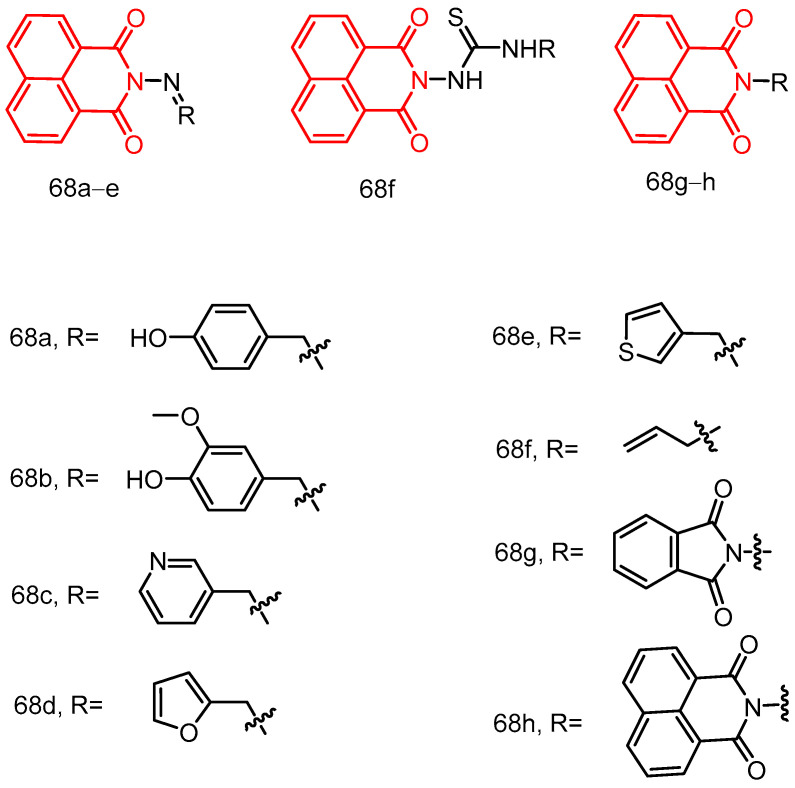
Chemical structures of the compounds **68a**–**h**.

In 2015, Al-Salahi et al. [98] reported a series of 2-aminonaphthalimide compounds (Figure 32) and evaluated their efficacy against herpes simplex virus HSV-1 and HSV-2. Their study represented the first investigation into the antiviral activity of this class of compounds. The newly synthesized series of 2-Aminobenzo[de]-isoquinoline-1,3-diones were assessed for their inhibitory effects against HSV-1 and HSV-2 using a cytopathic effect inhibition assay. Furaldehyde, thiophenal, and allyl isothiocyanide derivatives **68d**–**f** demonstrated potent inhibitory activity against HSV-1 (with effective concentration (EC_50_) values of 19.6, 16.2, and 17.8 μg/mL, respectively), while the control drug acyclovir exhibited an EC_50_ value of 1.8 μg/mL. In addition, compounds **68d** and **68e** showed promising activity against HSV-2. Several tested compounds displayed weak to moderate EC_50_ values compared to their inactive parent compound (2-Aminobenzo[de]-isoquinoline-1,3-diones), with compounds **68a**–**h** being identified as the most active antiviral agents in this series.

**Figure 33:**
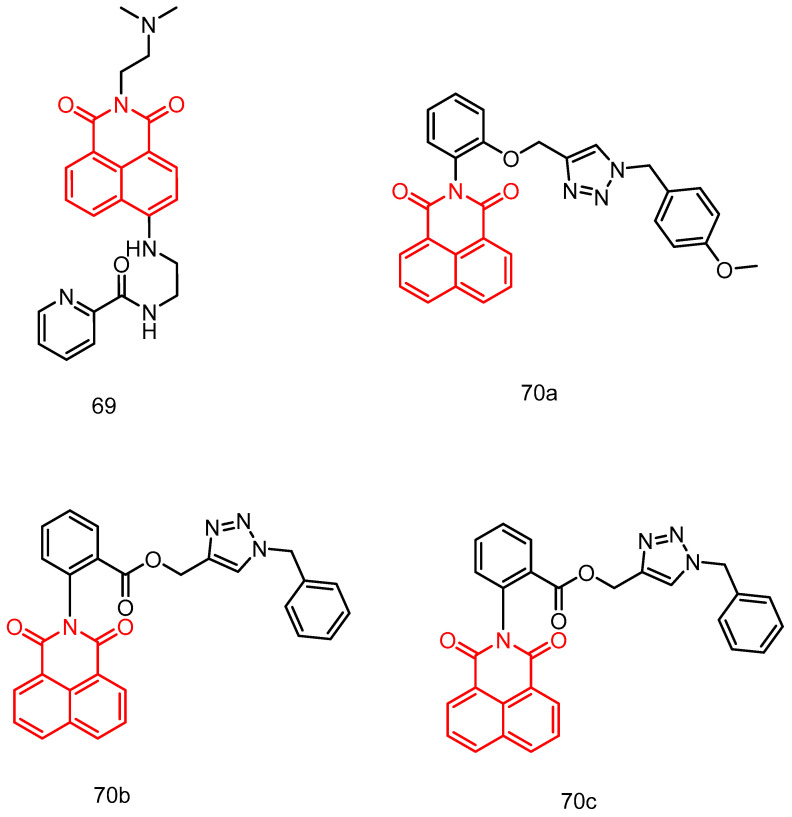
Chemical structures of the compounds **69**, **70a**–**c**.

In 2021, Shih et al. [99] reported a naphthylimide derivative, compound **69** (Figure 33) (IC_50_ = 5–10 μM), which inhibited collagen- and convulsin-mediated, but not thrombin or U46619-mediated, platelet aggregation. Compound **69** was more sensitive to the inhibition of glycoprotein VI (GPVI) signaling. Compound **69** inhibits the phosphorylation of signaling molecules downstream of GPVI, which in turn inhibits calcium mobilization, granule release, and GPIIb/IIIa activation. Compound **69** had a preventive effect on pulmonary embolism, with a prolonged blocking time, but tended to prolong the bleeding time. This suggests that compound **69** can prevent thrombosis but may increase the risk of bleeding.

In 2022, Begam et al. [100] reported novel heterocycles with 1,2,3-triazole derivatives as chain-linked naphthylimide groups. In vitro anti-inflammatory activity assays showed that compounds **70a**–**c** (Figure 33) exhibited significant and selective inhibitory effects at 200 μM. Compound **70b** inhibited the denaturation of bovine serum albumin and egg albumin by 92.3% and 95.29%, respectively. Compound **70b** exhibited higher inhibitory activity than **70a** and **70c**. Compound **70b** showed an inhibitory effect comparable to that of the standard drug diclofenac sodium. Molecular docking studies on COX1 and COX2 revealed the strong inhibitory activity of **70b**, with binding free energies of −13.58 and −10.42 kcal/mol, respectively, suggesting its potential as an anti-inflammatory agent.

**Table 1:** Antibacterial data as MIC for compounds.

Compound	Gram-Positive Bacteria	Activities as MIC (μg/mL)	Gram-Negative Bacteria	Activities as MIC (μg/mL)	Fungi	Activities as MIC (μg/mL)	Reference
**1a–d**	*S. aureus* MRSA *B. subtilis* *M. luteus*	≥256	*B. proteus* *E.* *coli* *P. aeruginosa* *B. typhi*	≥256	*C. albicans* *C. mycoderma*	≥256	[24]
**2a–i**		4–32		1–32		4–64	
**3a**		4–16		2–16		4–8	[21]
**3b**		8–16		4–16		8–16	
**3c**		4–8		2–8		8–16	
**3d**		8–16		2–16		16	
**3e**		4–512		4–8		8–16	
**3f**		2–512		2–4		4–8	
**3g**		2–512		2–4		4–8	
**4a**		14–29		14–512	*C. albicans* *C. mycoderma* *C. utilis* *S. cerevisiae* *A. flavus*	3.6–512	[20]
**4b**		2–14		7–14		2–14	
**4c**		7–230		115–230		7–29	
**4d**		57–205		205–512		4–14	
**5a**		57–115		57–205		57–512	
**5b**		2–29		19–29		512	
**5c**		19–115		57–230		29–115	
**5d**		230–512		230–512		205–512	
**6a**		>512		1–512	*C. albicans* *C. mycoderma*	64–512	[25]
**6b**		256–512		0.5–512		32–512	
**7**	*B. subtilis*	7.6	-	-	*C. albicans*	122	[26]
**8**	*-*	-	-	-	*C. albicans* *C. albicans* 9023 *Aspergillus fumigatus* *Candida tropicalis* *Candida parapsilosis*	0.772–64	[27]
**9**	*B. subtilis* *S. aureus* *E. coli*	12–24	*K. pneumoniae*	6.226	*C. albicans* *A. niger*	6.226–50	[28]
**10a**	*E. faecalis* *B. subtilis* *L. species* *S. aureus*	1.56–200	*S. enterica* *A. calcoaceticus* *S. marcescens*	100–200	*-*	-	[29]
**10b**		6.25–200		50–200			
**10c**		0.39–200		50–200			
**10d**		3.12–100		200			
**10e**		0.003–6.25		100–200			
**10f**		0.09–200		50–200			
**10g**		3.12–200		200			
**10h**		1.56–50		200			
**10i**		0.048–200		50–200			
**10k**		0.048–200		12.5–200			
**10l**		0.19–200		12.5–200			
**11a**	*S. aureus* *MRSA* *B. subtilis* *M. luteus*	0.04–0.08 µmol/mL	*B. typhi* *E. coli* (DH52)*E. coli* (**JM109**) *P. vulgarisP. aeruginosa* *S. dysenteriae*	0.01–0.16 µmol/mL	*C. albicans* *C. mycoderma* *B. yeast* *C. utilis* *A. flavus*	0.02–0.16 µmol/mL	[31]
**11b**		0.04–0.15 µmol/mL		0.002–0.15 µmol/mL		0.01–0.04 µmol/mL	
**11c**		0.02–0.07 µmol/mL		0.02–1.17 µmol/mL		0.02–0.29 µmol/mL	
**11d**		0.04–0.14 µmol/mL		0.02–1.13 µmol/mL		0.04–0.57 µmol/mL	
**11e**		0.91 µmol/mL		0.45–0.91 µmol/mL		0.23–0.91 µmol/mL	
**11f**		0.07–0.29 µmol/mL		0.04–0.29 µmol/mL		0.15–0.29 µmol/mL	
**11g**		0.07–0.29 µmol/mL		0.07–0.57 µmol/mL		0.07–0.28 µmol/mL	
**12a**	*S. aureus* *MRSA* *B. subtilis* *M. luteus*	0.2–0.8 µmol/mL	*B. typhi* *E. coli* (DH52) *E. coli* (**JM109**) *B. proteusP. aeruginosa* *S. dysenteriae*	0.01–0.8 µmol/mL	*C. albicans* *C. mycoderma* *B. yeast* *C. utilis* *A. flavus*	0.01–0.81 µmol/mL	[32]
**12b**		0.007–0.43 µmol/mL		0.11–0.43 µmol/mL		0.22–0.43 µmol/mL	
**12c**		0.003–0.85 µmol/mL		0.05–0.42 µmol/mL		0.003–0.42 µmol/mL	
**13**	*S. aureus* *B. subtilis* *B. cereus* *S. epidermis*	<0.65–2.5	*E. coli* *P. mirabilis*	<0.65	*-*	-	[33]
**14**	*S. aureus* MRSA *S. aureus* 25,923 *S. aureus* 29,213 *E. faecalis*	2–16	*K. pneumonia* *E. coli* *E. coli* 25,922 *A. baumanii* *P. aeruginosa* *P. aeruginosa* ATCC 27,853	4–128	*C. albicans* *C. albicans* *ATCC* 90,023 *C. tropicals* *A. fumigatus* *C. parapsilosis ATCC* 22,019	4–64	[38]
**15a**	MRSA *E. faecalis* *S. aureus* *S. aureus* ATCC 25,923 *S. aureus* ATCC 29,213	8–64	*K. pneumoniae* *E. coli* *P. aeruginosa* *A. baumanii* *P. aeruginosa* ATCC 27,853 *E. coli* ATCC 25,922	2–64	*-*	-	[39]
**15b**		8–128		2–64			
**15c**		1–64		2–64			
**15d**		4–64		2–64			
**15e**		4–64		2–128			
**16a**		2–64		16–64			
**16b**		4–64		4–128			
**16c**		8–64		8–128			
**16d**		0,5–16		2–16			
**17a**		1–128		16–64			
**17b**		2–64		16–64			
**17c**		1–128		1–128			
**17d**		1–64		4–32			
**17e**		1–128		16–128			
**17f**		8–16		8–256			
**18a**		2–64		1–128	*-*	-	[40]
**18b**		2–32		4–64			
**18c**		1–64		2–64			
**18d**		4–64		2–64			
**18e**		0.5–64		1–64			
**18f**		32–128		0.5–64			
**18g**		0.25–64		2–64			
**18h**		2–64		4–64			
**18i**		0.5–16		4–64			
**18j**		0.25–64		4–64			
**18k**		4–128		0.5–64			
**18m**		0.25–64		2–128			
**18l**		4–128		4–64			
**19a**	*S. aureus* ATCC 29,213	2	Mtb H37Rv ATCC 27,294	>64	MRSA ATCC 29,213 MRSA NRS 100 MRSA NRS 119 MRSA NRS 129 MRSA NRS 186 MRSA NRS 191 MRSA NRS 192 MRSA NRS 193 MRSA NRS 194 MRSA NRS 198 VRSA VRS 1 VRSA VRS 4 VRSA VRS 12	2–4	[41]
**19b–g**		>64		>64		0.25–0.5	
**19h**		0.125		4		-	
**19i**		8		32		-	
**20a–k**		1–>64		8–>64		-	
**20f**		0.25		>64		0.125–0.5	
**20l**		1		>64		1–4	
**21a**		0.25		>64		0.125–0.5	
**21b**		0.03125		>64		0.25–1	
**23**	*-*	-	*-*	-	*C. albicans* *C. albicans* ATCC 90,023 *C. tropicals* *A. fumigatus* *C. parapsilosis* ATCC 22,019	4–128	[43]
**25a**	*S. aureus* ATCC 29,213	4	*E. coli* ATCC 25,922 *K. pneumoniae* BAA 1705 *A. baumannii* BAA 1605 *P. aeruginosa* ATCC 27,853	>64	Mtb H37Rv ATCC 27,294	16	[45]
**25b**		2		>64		16	
**25c**		2		>64		>64	
**25d**		2		>64		16	
**25e**		4		>64		32	
**25f**		1		>64		32	
**25g**		0.5		>64		64	
**25h**		2		>64		>64	
**25i**		>64		>64		1	
**25j–o**		>64		>64		8–>64	
**25p–q**		>64		>64		>64	

**Table 2:** Anticancer data as MIC for compounds.

Compound	Cancer Cell Lines	IC_50_/GI_50_ Values (µM)	Reference
**26**	Hs683, U373MG, HCT-15, LoVo, A549, MCF-7	0.8–1.8	[64]
**27**	PC-3, DU-145, U373-MG, Hs683, HCT-15, LoVo, MCF-7, A549, Bx-PC-3	4.7–46.5	[65]
**28a**	HeLa, P388D1	0.62 ± 0.07–0.83 ± 0.08	[66]
**28b**		0.23 ± 0.07–0.71 ± 0.05	
**28c**		0.43 ± 0.09–1.93 ± 0.06	
**29**	SK-OV-3, HepG2, A-549, T-24 SMMC-7721, HL-7702	4.13 ± 0.9–20.71 ± 2.1	[67]
**30a**	A549, A549R, NB-4, A261, HLF	1.5 ± 0.1–9.9 ± 0.1	[68]
**30b**		2.9 ± 0.04–12.9 ± 0.02	
**30c**		4.1 ± 0.03–8.4 ± 0.2	
**31a**	HeLa, HepG2, A549	5.06 ± 0.25–20.26 ± 0.30	[69]
**31b**		8.48 ± 0.20–38.27 ± 0.26	
**31c**		17.02 ± 0.16–78.66 ± 0.20	
**31d**		4.85 ± 0.16–30.95 ± 0.17	
**31e**		8.95 ± 0.25–74.30 ± 0.12	
**31f**		24.19 ± 0.11–155.13 ± 0.04	
**31g**		34.81 ± 0.10–264.17 ± 0.07	
**31h**		16.74 ± 0.18–39.92 ± 0.13	
**32a**	Jurkat, HeLa, MCF-7, A-549	6.53 ± 0.48–>50	[70]
**32b**		5.67 ± 0.12–>50	
**33**	HCT-116, HepG2, K562, MDA-MB-231, QSG-7701	2.86–53.85	[71]
**34**	K562, HepG2, HCT116, SMMC-7721	3.30 ± 1.01–18.95 ± 2.17	[72]
**35**	K562, HepG2, HCT116, SMMC-7721	4.67 ± 0.87–6.34 ± 1.41	[73]
**36a**	Snu-368, Snu-739, MDA-MB-231 MCF-7, A549, A549cisR	1.19 ± 0.12–3.62 ± 0.15	[74]
**36b**		1.07 ± 0.10–3.89 ± 0.35	
**36c**		0.83 ± 0.08–1.92 ± 0.23	
**37**	HepG-2, Huh-7, MDA-MB-231, MCF-7 A549, A549cisR	0.98 ± 0.19–20.32 ± 0.78	[75]
**38a**	Hela, MCF-7, A549, MGC-803	0.60 ± 0.10–37.07 ± 0.04	[76]
**38b**		2.03 ± 0.25–22.70 ± 0.02	
**38c**		6.88 ± 0.05–90.43 ± 0.2	
**38d**		1.60 ± 0.37–20.01 ± 0.0	
**38e**		5.96 ± 0.08–141.7 ± 0.02	
**38f**		9.41 ± 0.27–74.63 ± 0.05	
**38g**		6.41 ± 0.34–93.53 ± 0.08	
**38h**		35.05 ± 0.13–146.52 ± 0.0	
**39a**	A549, MRC-5I	0.51 ± 0.13–1.30 ± 0.23	[77]
**39b**		0.15 ± 0.03–0.95 ± 0.19	
**39c**		0.89 ± 0.07–1.35 ± 0.43	
**39d**		1.04 ± 0.07–2.22 ± 0.49	
**40**	A549, MCF-7, PC-3, Hela, RPE1	7.6 ± 0.78–>50	[78]
**41**	EC109, BGC823	0.07799–0.14245	[79]
**42a**	HT-29, A549, MCF-7	03.72 ± 0.3–07.91 ± 0.4	[80]
**42b**		03.47 ± 0.2–05.08 ± 0.3	
**43a**	HL-60, MCF-7, HepG,2 HeLa, SK-OV-3 MT-4, LO2, BEAS-2B, SH-SY5Y	46.79 ± 1.96–>100	[81]
**43b**		14.66 ± 0.31–>100	
**44a**	Leukemia, non-small cell lung cancer, colon cancer, CNS cancer, melanoma, ovarian cancer, renal cancer, prostate cancer, breast cancer.	GI_50_ = 1.09–1.67	[82]
**44b**		GI_50_ = 1.21–2.68	
**45a**	MDA-MB-231, HepG-2, PC12, A549	10.86–>100	[26]
**45b**		2.2699–91.453	
**46**	A549, SK-OV-3, HT-29, HL-60, PC-3, HepG2, MDA-MB-231, MRC-5	6.73 ± 0.37–14.00 ± 0.56	[83]
**47a**	A549, HL-60	3.1–7.1	[49]
**47b**		1.9–2.5	
**47c**		1.4–3.6	
**48a**	HepG2, SK-N-SH, MCF-7, PC-3, AGS, A549, MDA-MB-231, K562, A375, 786-O, SH-SY5Y, BE(2)-M17, SK-N-AS, IMR-32	0.59 ± 0.08–2.75 ± 0.06	[84]
**48b**		0.80 ± 0.06–4.232 ± 0.07	
**49a**	T24, MGC-803, CNE-2, A2780	0.09 ± 0.06–1.24 ± 0.12	[85]
**49b**		1.48 ± 0.12–4.81 ± 0.22	
**50**	Leukaemia, non-small cell lung cancer, colon cancer, CNS cancer, melanoma, ovarian cancer, renal cancer, prostate cancer, breast cancer. a	GI_50_ = 2.9–6.97	[86]
**51**	HepG2	34.2	[87]
**52a**	A549, HeLa, HepG2, Beas-2b	11.3 ± 0.1–15.6 ± 1.2	[88]
**52b**		4.9 ± 0.7–17.1 ± 3.6	
**53**		10.4 ± 1.3–19.3 ± 6.0	
**54a**	EC109, BGC823, SGC 7901, HePG2	>200	[89]
**54b**		68.54–123.9	
**55**		4.33–10.52	
**56a**	MCF-7, HT-29	4.6 ± 3.6–27.5 ± 16.2	[90]
**56b**		1.9 ± 0.4–16.0 ± 0.7	
**56c**		1.5 ± 0.3–9.6 ± 0.2	
**57a**		5.8 ± 0.5–10.0 ± 0.4	
**57b**		1.7 ± 0.3–6.5 ± 0.4	
**57c**		4.0 ± 0.4–6.2 ± 0. 4	
**58a**		18.6 ± 1.3–36.8 ± 0.48	
**58b**		11.6 ± 1.0–26.4 ± 1.1	
**58c**		4.8 ± 0.1–4.9 ± 0.02	
**59a**	SKOV-3, A549, A549R, HeLa Hela/DDP	8.64 ± 1.41–44.47 ± 12.44	[91]
**59b**		1.47 ± 0.11–14.94 ± 1.20	
**59c**		3.1 ± 0.43–71.74 ± 10.65	
**59d**		2.49 ± 0.30–35.35 ± 2.51	
**59e**		1.28 ± 0.28–6.53 ± 2.90	
**60**	HT-29, HCT-116, MDA-MB-231, MCF-7, 4T1, A549cisR, A549	1.45 ± 0.45–14.17 ± 1.37	[92]
**61a**	Hela, HepG-2, NCI-H460, BEL-7404, SMMC-7721, U251	6.85 ± 1.41–78.30 ± 1.32	[93]
**61b**		5.46 ± 0.76–>100	
**61c**		4.92 ± 1.55–>100	
**61d**		4.99 ± 1.64–>100	
**61e**		2.36 ± 1.44–>100	
**61f**		7.33 ± 0.89–>100	
**62a**	SK-OV-3, NCI-H460, HeLa, HL-7702	11.32 ± 0.47–45.07 ± 0.37	[94]
**62b**		0.89 ± 0.25–50.22 ± 1.04	

## 3. Conclusions and Prospects

In conclusion, heterocyclic naphthalimides, a novel class of backbone structural compounds, have garnered significant attention for their rapid development and remarkable pharmacological activities. This paper presents a comprehensive review of the potential applications of naphthylimide derivatives in antibacterial, antifungal, anticancer, antiviral, anti-inflammatory, antiprotozoal, antithrombotic, and antimalarial therapies. The increasing prevalence of drug-resistant and refractory pathogenic microorganisms, coupled with the emergence of new pathogens, poses a growing and challenging public health problem globally. Numerous studies have focused on developing multi-targeted active molecules derived from naphthylimine analogues. It has been reported that a number of naphthylimide analogs have been identified as clinical candidates, due to their good tolerability, broad activity spectrum, low toxicity, high bioavailability, and favorable pharmacokinetic properties. This review underscores the considerable potential of naphthylimide analogues for treating antibacterial and antifungal infections. The planar and heteroaromatic nature of naphthylimides enables strong DNA intercalation, making these derivatives valuable in anticancer therapies. The presence of *N*,*N*-diethylethylenediamine is indispensable for their antiproliferative properties, and the substitution pattern on the ring nitrogen significantly impacts these properties. A number of novel and potent molecules have emerged as drug precursors, with some already undergoing preclinical studies. Meanwhile, the exploration of other biological properties associated with these molecules has prompted further investigation into the diverse characteristics of naphthylimide derivatives. The other pharmacological attributes of this important structural class remain to be elucidated. With the influx of new molecules, it is anticipated that more potent drugs will emerge in the near future. Enhancing the water solubility of these derivatives is pivotal for drug development and should be a focal point for future research efforts. Examples of drugs containing naphthylimides and their pharmacological applications can also be found in current literature.

## Data Availability

There are no data associated with this publication.
